# Outcomes of post-approval non-interventional safety studies in pregnancy: wide variation demonstrates need for further standardization

**DOI:** 10.3389/fdsfr.2026.1805759

**Published:** 2026-05-11

**Authors:** Stacy Chen, Sarah C. MacDonald

**Affiliations:** 1 Safety Surveillance Research, Worldwide Medical and Safety, Pfizer, Inc., New York, NY, United States; 2 Safety Surveillance Research, Worldwide Medical and Safety, Pfizer, Inc., Ottawa, ON, Canada

**Keywords:** outcomes, post-approval studies, pregnancy, regulatory guidance, safety

## Abstract

**Background:**

Upon product approval, sponsors are often required by regulatory authorities (e.g., the United States [US] Food and Drug Administration [FDA] or the European Medicines Agency [EMA]) to conduct post-approval safety studies in pregnant populations. Existing guidance from regulatory authorities provide important recommendations of study outcomes to evaluate in such studies, however harmonized guidance on a priority list of outcomes that all studies must evaluate is currently lacking. This creates inefficiencies in study conduct and limits comparability.

**Objectives:**

The article aims to summarize outcomes that are currently being studied across post-approval safety studies in pregnancy and assess the influence of study-related factors on the evaluated outcomes.

**Methods:**

A query of post-approval non-interventional pregnancy safety studies registered on the Heads of Medicines Agency [HMA]-EMA Catalogue of Real-world Data [RWD] Studies was conducted, and relevant studies and their protocols were extracted for evaluation. Outcomes were summarized overall and by factors such as data collection method, product type, protocol year, and regulatory agency commitment.

**Results:**

Among approximately 3100 studies identified in the catalogue, 30 studies met eligibility criteria for inclusion. The most common outcomes were stillbirth (N = 25 studies), congenital malformations (major or minor) (N = 24), spontaneous abortion (N = 22), small for gestational age or fetal growth restriction (N = 21), and preterm birth or labor (N = 21). Some differences in evaluated outcomes were observed by study characteristics (e.g., regulatory agency commitment, data collection methods, type of active substance, date of protocol). No outcomes were evaluated in all of the studies and none of the studies evaluated all FDA or EMA-recommended outcomes. Sample sizes from completed studies were generally low (median: 110) and few studies had sufficient power to examine commonly evaluated outcomes such as stillbirth and congenital malformations.

**Conclusion:**

There was a wide variety of outcomes examined across post-marketing safety studies in pregnancy. Additional harmonized regulatory guidance on required outcomes will improve consistency across studies, allowing for meaningful comparisons and for the more efficient use of existing data.

## 1 Introduction

As pregnant populations are typically excluded from clinical trials, sponsors may be required to conduct at least one non-interventional safety study in pregnancy upon product approval (United States Food and Drug Administration, 2019). Depending on the product, these studies can last a long time and be costly to conduct for, what is often, a limited sample size. For example, a previous systematic review that examined pregnancy registries for drug or biologic products reported the median enrollment to be only 36 pregnancies (Bird et al., 2018).

While the products themselves range in indication, the selection of study outcomes is often indication independent. Indeed, many outcomes are pregnancy-specific (e.g., major congenital malformations, stillbirth) and applicable to a range of study products and indications. While existing guidelines from the United States (US) Food and Drug Administration (FDA) and European Medicines Agency (EMA) (United States Food and Drug Administration, 2019; European Medicines Agency, 2019; European Medicines Agency, 2005; The European Network of Centres for Pharmacoepidemiology and Pharmacovigilance, 2025), provide important recommendations of study outcomes that can be considered, neither authority provides clear guidance on a priority list of outcomes that *all* studies should evaluate. Sponsors are therefore left to propose a set of outcomes without clear justification until further feedback is received from the regulatory agency (either in the Post-marketing Requirement letter or during protocol review). However, this is inefficient and can result in different outcomes being evaluated across studies, which limits comparability. Although a recent review of selected post-approval safety studies in pregnancy for vaccine products found substantial variation in outcomes that were evaluated (MacDonald et al., 2025), this assessment was not systematic, nor did it provide information about factors that may influence the choice of study outcomes.

This research therefore builds upon prior findings to systematically summarize outcomes that are studied across post-approval safety studies in pregnancy and assess the influence of study-related factors on the evaluated outcomes.

## 2 Methods

The Heads of Medicines Agency [HMA]-European Medicine Agency [EMA] Catalogue of Real-world Data [RWD] Studies was queried on 12 June 2025 with a focus on non-interventional post-approval safety studies among pregnant people. The following inclusion criteria were applied on the website filters:

- Special population group = “Pregnant Women”.
- Study Type = “Non-interventional Study”.
- Study required by a Regulator = “Yes”.
- Scope of the Study = “Safety study (incl. comparative)”.
- Status of Research = “Finalized” or “Ongoing” or “Planned”.
- Study Topic = “Human Medicinal Product”.

Studies were excluded if:

- Status of Research = “Discontinued” or “Cancelled”.
- Lack of publicly available protocol.
- No prespecified safety outcome.
- Relied on only spontaneous case report data.
- Lack of an exposed medicinal or vaccinated cohort.

Among identified studies, the following information was collected from their publicly available protocols and reports, if available:

- Status of Research.
- Initial, Most Recent, and Final Protocol Year (if applicable).
- Start and End of Study Period.
- Regulatory Requirement (either FDA, EMA or a different agency).
- Active Substance (including if it was a drug or vaccine).
- Study Design and Main Data Collection Method (primary data collection vs. secondary data use).
- Data Source.
- Final/Target Sample Size for Exposed group.
- Outcomes (including if a chart validation was performed).

Outcomes were recorded by name as they were listed in the protocol and then consolidated by the authors into common groups of similar outcomes. The mapping between the protocol-listed outcome name and the consolidated outcome name can be found in Supplementary Table S1 in Supplementary Material.

This review is a descriptive analysis. Study characteristics were summarized, including available or target sample size and the use of chart validation. Outcomes were summarized overall across studies and by the following key factors: regulatory agency commitment (FDA or EMA), product type (medicinal or vaccine), data collection methods (primary or secondary), and year of protocol development. Percentages and 95% confidence intervals were calculated.

## 3 Results

**FIGURE 1:**
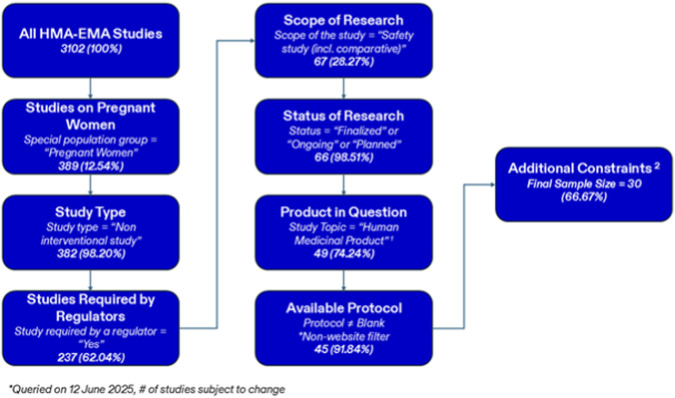
Flowchart for Inclusion Criteria Abbreviations: HMA-EMA = Heads of Medicines Agencies-European Medicine Agency The focus is to limit the study criteria to strictly non-interventional safety studies in pregnant populations that were required by regulators. 1 Examples of study topics that were excluded would be herbal medicinal product, medical device or medical procedure. 2 Additional constraints included that the study must explicitly state a type of safety outcome to be measured, and exclusion of studies relying on data from only spontaneous-case reports with no prespecified outcome. One study was excluded due to lack of exposed medication group.

Of the 3102 studies available on the HMA-EMA catalogue at the time of query, 389 (12.54%) were indicated on the website as including pregnant women (Figure 1). Of those, most (N = 382 [12.31%]) were non-interventional and required by a regulator (N = 237 [7.64%]). Approximately a third of the studies evaluated safety (N = 67 [2.16%]) and most were ongoing, finalized, or planned (N = 66 [2.13%]). A total of 49 studies evaluated a human medicinal product (N = 49 [1.58%]) and 45 (1.45%) had an available protocol to evaluate. After all additional constraints (including requiring the study to have prespecified safety outcomes, excluding studies relying only on spontaneous case report data, and requiring the study to have an exposure cohort) were applied, 30 (0.97%) studies were included in the final cohort.

Supplementary Tables S2, S3 provide an overview of the available studies, including the year of their most recent protocol, main data collection method, their target sample size/enrolment size, final sample size if applicable, and a list of outcomes.

Of the included studies, 15 (50.00%) studies were indicated on the website as finalized, 8 (26.67%) were ongoing, and 7 (23.33%) were planned. The earliest study protocol was dated in 2013, and the latest study protocol was dated in 2025. A total of 7/16 (43.75%) secondary studies stated that a chart review would be performed. A total of 7/16 (43.75%) secondary studies did not state that a chart review would be performed, however the data was recorded directly by physicians, from medical charts, or previous validations had been conducted for some outcomes. Only 2/16 (12.50%) secondary studies did not perform a chart validation and did not meet the above criteria.

Generally, studies that were planned or ongoing targeted more exposed patients than the final sample size of the finalized studies (Median of planned/ongoing studies: 417; median of finalized studies: 110) (Table 1). Indeed, while a substantial proportion of finalized studies included only 0-50 exposed patients (6/15; 40.00%), only 1 of 12 (8.33%) planned or ongoing studies targeted such a small cohort. Similarly, planned or ongoing studies were more likely than finalized studies to target or include over 100 exposed patients (ongoing/planned studies: 83.34% vs. finalized studies: 59.97%).

**TABLE 1:** Sample size summary.

Summary Statistics	Final sample size of exposed cohorts (finalized studies) N = 15	Targeted sample size of exposed cohort (planned or ongoing studies) N = 12^a^
Min-max	0-3515	15-1152
Mean (SD)	906.60 (1317.83)	504.08 (360.46)
Median	110	417
Proportion of studies with		
0-50	6/15 (40.00%)	1/12 (8.33%)
50-<100	0/15 (0.00%)	1/12 (8.33%)
100-<500	3/15 (20.00%)	5/12 (41.67%)
500-<1000	2/15 (13.33%)	3/12 (25.00%)
1000+	4/15 (26.67%)	2/12 (16.67%)

^a^ There were 15 total planned or ongoing studies, but some studies lacked a target enrollment group or there was no single target and thus excluded from the calculations.

The most frequently studied outcomes include stillbirth (N = 25 studies), congenital malformations (major or minor) (N = 24), spontaneous abortion (N = 22), small for gestational age or fetal growth restriction (N = 21), preterm birth or labor (N = 21), elective termination (N = 15), and preeclampsia (N = 13). Table 2 lists all consolidated outcomes that were evaluated at least twice. Outcomes that were only evaluated once were not summarized in the tables.

**TABLE 2:** List of all outcomes measured and frequency.

Outcome categories	Specified outcome	Count^a^	% Of total studies N = 30	95% CI
Pregnancy	Stillbirth	25	83.33%	[70.00%, 96.67%]
	Spontaneous abortion	22	73.33%	[57.51%, 89.16%]
	Preterm birth or labor	21	70.00%	[53.60%, 86.40%]
	Elective termination	15	50.00%	[32.11%, 67.89%]
	Ectopic pregnancy	3	10.00%	[0.00%, 20.74%]
	Fetal loss (type not specified)	2	6.67%	[0.00%, 15.59%]
	Termination of pregnancy for fetal anomaly (TOPFA)	2	6.67%	[0.00%, 15.59%]
	Molar pregnancy	2	6.67%	[0.00%, 15.59%]
	Premature rupture of membranes (PROM)	2	6.67%	[0.00%, 15.59%]
	Preterm PROM (PPROM)	2	6.67%	[0.00%, 15.59%]
	Placental conditions	2	6.67%	[0.00%, 15.59%]
Maternal	Preeclampsia	13	43.33%	[25.60%, 61.07%]
	Gestational hypertension	8	26.67%	[10.84%, 42.49%]
	Gestational diabetes	8	26.67%	[10.84%, 42.49%]
	Eclampsia	7	23.33%	[8.20%, 38.47%]
	Caesarean delivery	7	23.33%	[8.20%, 38.47%]
	Guillain-Barré Syndrome	3	10.00%	[0.00%, 20.74%]
	Maternal death	3	10.00%	[0.00%, 20.74%]
	Atrial fibrillation	2	6.67%	[0.00%, 15.59%]
	Postartum hypertension	2	6.67%	[0.00%, 15.59%]
	Chronic hypertension superimposed with preeclampsia/eclampsia	2	6.67%	[0.00%, 15.59%]
	Maternal length of stay	2	6.67%	[0.00%, 15.59%]
	Polyneuropathies	2	6.67%	[0.00%, 15.59%]
	Immune-mediated demyelinating conditions	2	6.67%	[0.00%, 15.59%]
	Thrombocytopenia	2	6.67%	[0.00%, 15.59%]
	HELLP Syndrome	2	6.67%	[0.00%, 15.59%]
Infant	Congenital malformations (major or minor)	24	80.00%	[65.69%, 94.31%]
	Small for gestational age or fetal growth restriction	21	70.00%	[53.60%, 86.40%]
	Infant growth and development	11	36.67%	[19.42%, 53.91%]
	Low birth weight	7	23.33%	[8.20%, 38.47%]
	Infant/Neonatal death	7	23.33%	[8.20%, 38.47%]
	Apgar score	5	16.67%	[3.33%, 30.00%]
	Admission to NICU	4	13.33%	[1.17%, 25.50%]
	Infant/neonatal infections or other illness	4	13.33%	[1.17%, 25.50%]
	Neurodevelopmental outcomes	3	10.00%	[0.00%, 20.74%]
	Infant hospitalization	3	10.00%	[0.00%, 20.74%]
	Large for gestational age	2	6.67%	[0.00%, 15.59%]

^a^ Outcomes that only had one occurrence were excluded from this table.

### 3.1 Regulatory commitment

Of the 30 studies evaluated, 16 (53.33%) were committed to the EMA whilst 14 (46.67%) were committed to the FDA (Supplementary Table S4). There were 4 (13.33%) studies that were required by different regulatory agencies: the Medicines and Healthcare products Regulatory Agency [MHRA], China Center for Drug Evaluation [CDE], Japan Pharmaceutical Affairs Law and Good Post-marketing Study Practice, and Agenzia Italiana del Farmaco.

**FIGURE 2:**
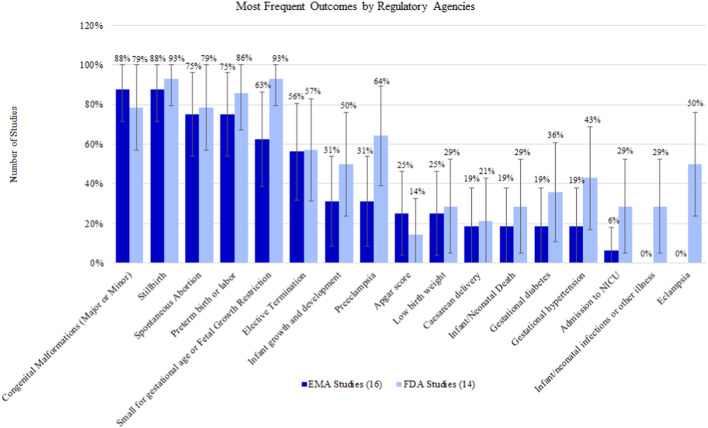
Percentage (95% confidence interval) of outcomes by regulatory agency commitments.

Among FDA-committed studies, the most studied outcome was stillbirth (13/14 studies [92.86%]) (Figure 2). Among the most frequently studied outcomes (defined as outcomes studied in at least two studies), low frequency outcomes included Apgar score (2/14 [14.29%]) and Caesarean delivery (3/14 [21.43%]), among others. There were no outcomes measured in all the FDA-committed studies. The average number of evaluated outcomes was approximately 12 per study (range: 5-25).

Among EMA-committed studies, stillbirth and congenital malformations (major or minor) were the most common outcomes, both appearing in 14/16 studies (87.50%). Eclampsia was not evaluated in any of the studies. There were no safety outcomes that were measured in all EMA-committed studies. The average number of evaluated outcomes was approximately seven per study (range: 2-11).

Most outcomes were measured at similar frequencies across EMA and FDA studies; however, some outcomes were more frequently captured in FDA-committed studies. These included: eclampsia (FDA studies: 50.00% vs. EMA studies: 0.00%), preeclampsia (64.29% vs. 31.25%), and small for gestational age or fetal growth restriction (92.86% vs. 62.50%).

### 3.2 Product type

Of the 6 studies that investigated vaccine safety, all evaluated stillbirth (Supplementary Table S5). This is followed by small for gestational age or fetal growth restriction and preterm birth or labor, both appearing in 5/6 studies (83.33%). Elective termination, Apgar score, and infant/neonatal infections or other illness were not measured in any vaccine studies.

Among studies evaluating medication safety (N = 24), the most common outcome was congenital malformations (major or minor) which was observed in 21/24 studies (87.50%), followed by spontaneous abortion appearing in 20/24 studies (83.33%), and stillbirth in 19/24 studies (79.17%). Low frequency outcomes in medicine studies included low birth weight (4/24 studies [16.67%]), infant/neonatal death (4/24 studies [16.67%]), infant/neonatal infections or other illness (4/24 studies [16.67%]), and admission to the NICU (2/24 studies [8.33%]) studies. There were no safety outcomes that were measured in all medicinal studies.

**FIGURE 3:**
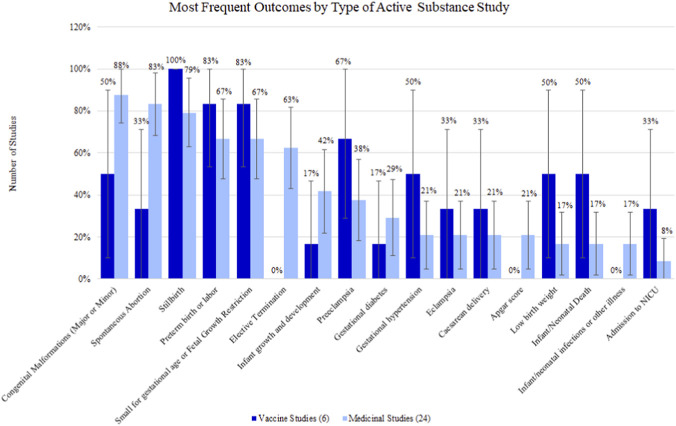
Percentage (95% confidence interval) of outcomes by type of active substance study.

In general, studies evaluating medicinal products tended to be slightly more likely than the vaccine studies to evaluate outcomes requiring early pregnancy exposures, including congenital malformations (medicinal: 87.50% vs. vaccine: 50.0%), spontaneous abortion (83.33% vs. 33.33%), and elective termination (62.50% vs. 0.00%). Outcomes that tended to be more common in vaccine studies included stillbirth (vaccine: 100.00% vs. medicinal: 79.17%), preeclampsia (66.67% vs. 37.50%), and gestational hypertension (50.00% vs. 20.83%), among others (Figure 3).

### 3.3 Data collection method

There were 14 (46.67%) studies that utilized primary data collection and 16 (53.33%) that utilized secondary data sources (Supplementary Table S6). Across 14 primary data collection studies, spontaneous abortion was evaluated the most frequently, appearing in 12/14 (85.71%) studies followed closely by congenital malformations (major or minor) and infant growth and development, both evaluated in 11/14 (78.57%) studies. Low birth weight was a low-frequency outcome, present in 2/14 (14.29%) studies.

Across 16 secondary data studies, stillbirth (93.75%) was the most evaluated outcome. Infant growth and development and infant/neonatal infections or other illness were not evaluated in any secondary database study.

**FIGURE 4:**
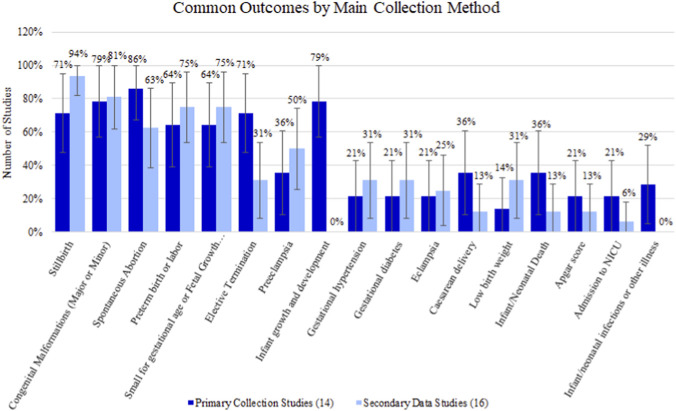
Percentage (95% confidence interval) of outcomes by main method of data collection.

Most outcomes occurred at a similar frequency across primary and secondary forms of data collection (Figure 4). However, studies using primary data collection were more likely than studies using secondary databases to evaluate infant growth and development (primary data collection: 78.57% vs. secondary databases: 0.00%) and elective termination (71.43% vs. 31.25%).

### 3.4 Year of protocol

**FIGURE 5:**
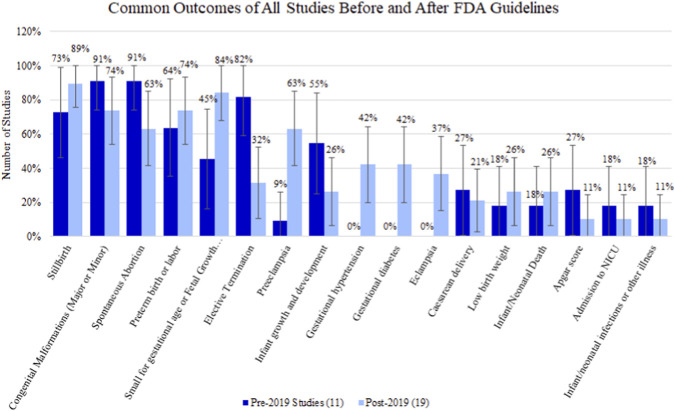
Percentage (95% confidence interval) of outcomes for all studies before and after FDA guideline implementation in 2019.

Across all studies (including non-FDA committed studies), there were 11 (36.67%) protocols dated prior to the FDA guidance implementation in May of 2019 (United States Food and Drug Administration, 2019) and 19 protocols (63.33%) dated after this date (Supplementary Table S7). Out of all studies, 9 outcomes saw a slight increase in percentage of evaluation after guidance (Supplementary Table S7). These outcomes included stillbirth, preterm birth or labor, small for gestational age or fetal growth restriction, preeclampsia, gestational hypertension, gestational diabetes, eclampsia, low birth weight, and infant/neonatal death (Figure 5).

All 4 FDA-committed studies written before FDA guidelines implementation in 2019 evaluated the outcomes of congenital malformations (major or minor), spontaneous abortion, and elective termination. None of these studies evaluated preeclampsia, gestational diabetes, eclampsia, gestational hypertension, and infant/neonatal death (Supplementary Table S8).

**FIGURE 6:**
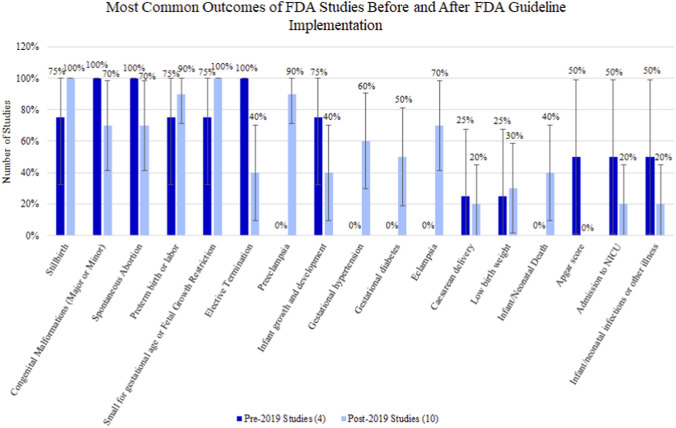
Percentage (95% confidence interval) of outcomes of FDA studies before and after FDA guideline implementation in 2019.

There were 10 FDA-committed studies with protocols dated after May 2019. A total of 9/17 outcomes saw an increase in percentage of evaluation (Figure 6), including preeclampsia (pre-2019: 0.00% vs. post-2019: 90.00%), gestational hypertension (0.00% vs. 60.00%), gestational diabetes (0.00% vs. 50.00%), eclampsia (0.00% vs. 70.00%), and infant/neonatal death (0.00% vs. 40.00%).

Some evaluated outcomes decreased in frequency after guideline implementation, including congenital malformations (major or minor) (100.00% vs. 70.00%), spontaneous abortion (100.00% vs. 70.00%), elective termination (100.00% vs. 40.00%), infant growth and development (75.00% vs. 40.00%), Apgar score (50.00% vs. 0.00%), admission to NICU (50.00% vs. 20.00%), and infant/neonatal infections or other illness (50.00% vs. 20.00%). However, sample size of pre-2019 dated studies was small (N = 4).

## 4 Discussion

This study aimed to provide an overview of existing non-interventional post-approval pregnancy safety studies that are commitments to regulatory agencies to assess concordance and determine factors that may influence the types of outcomes measured. Overall, 30 studies were evaluated. Across these studies, there was a wide variation of outcomes measured. The most common outcomes were stillbirth, congenital malformations (major or minor), spontaneous abortion, small for gestational age or fetal growth restriction, and preterm birth or labor. Differences in evaluated outcomes were observed by study characteristics, including data collection method, regulatory commitment agency, and type of active substance. In accordance with existing guidelines, most secondary data studies conducted chart review or used data recorded directly by physicians, from medical charts, or using algorithms that had previously validated for at least some outcomes.

As mentioned, the FDA draft guidelines (United States Food and Drug Administration, 2019), released in 2019, do not explicitly state what types of outcomes studies *must* assess. However, a list of recommended data collection elements are provided which includes the outcome of pregnancy (e.g., livebirth, stillbirth, spontaneous abortion, elective termination, preterm birth or labor), gestational complications (e.g., preeclampsia, eclampsia, gestational hypertension, gestational diabetes), infant and neonatal outcomes (e.g., anomalies diagnosed at birth or termination [including autopsy results], anomalies diagnosed after birth, weight at birth indicating whether small, appropriate, or large for gestational age, length at birth, head circumference at birth, condition at birth [including, when available, Apgar scores at 1 and 5 min, need for resuscitation, admission to intensive care nursery, neonatal illnesses, hospitalizations], and infant developmental milestones). In this analysis, none of the evaluated studies, including FDA-committed studies among protocols dated after the guidance was established, examined all of these recommended outcomes. The reasons for this are unknown but may be related to product-specific study needs and/or limitations of the selected data source or registry.

Guidance from the EMA states that studies should explicitly address and justify which pregnancy and infant/child outcomes will be evaluated (European Medicines Agency, 2019), however the existing EMA guidance (European Medicines Agency, 2019; European Medicines Agency, 2005; The European Network of Centres for Pharmacoepidemiology and Pharmacovigilance (ENCePP), 2025) does not provide a comprehensive list of outcomes that sponsors should evaluate. Nevertheless, there are some listed outcomes of interest. These include pregnancy outcomes (e.g., spontaneous abortion, elective termination, termination of pregnancy due to fetal anomaly, ectopic pregnancy, molar pregnancy, stillbirth), maternal outcomes (e.g., preeclampsia, gestational diabetes, maternal death, placental conditions), and infant, fetal and neonatal outcomes (e.g., congenital malformations (major or minor), low birth weight, small for gestational age, Apgar score, neonatal dysmaturity, neonatal illness). Long-term developmental milestones for neurodevelopmental disorders are also noted. Like the FDA guidance, none of the evaluated studies in this analysis examined all the outcomes listed by the EMA. Long-term neurodevelopmental outcomes (defined in this analysis as conditions such as attention deficit hyperactivity disorder, autism spectrum disorders, and learning disabilities) were of particularly low occurrence, appearing in only 3 studies. This may be due to limitations in ascertaining these outcomes in the data, issues of loss to follow-up, and/or study time restrictions.

Further, existing guidance documents provide little differentiation between outcomes to be evaluated in secondary database studies vs. studies using primary data collection (e.g., pregnancy registries). In our analysis, the listed outcomes were similar across primary vs. secondary data studies, with a few exceptions, mainly infant growth and development and elective termination, which were more common in primary data collection studies. These outcomes can be difficult to capture in secondary data sources where certain growth metrics like weight, height, and head circumference are rarely included. FDA guidance (United States Food and Drug Administration, 2019) notes, “Direct access to the mothers allows specialized physical examinations and developmental follow-up of the offspring.” Thus, developmental milestones may be difficult to capture if the studies are using secondary data as opposed to primary. The difference in elective termination may be due to the type of secondary data source. Medical charts may be more likely to indicate elective termination on a patient’s file. On the contrary, there is a chance that certain insurances may not cover elective terminations or women may choose to pay the costs out of pocket, thus not appearing in a claims database (Jones et al., 2013). The significant overlap between outcomes measured across data collection methods raises the question of efficiency, particularly for sponsors required to conduct studies in both primary and secondary data sources. While the FDA’s guidance indicates that all the recommended data collection elements apply also to studies using complementary (i.e., non-registry) designs, not all the stated outcomes (e.g., head circumference) are well-suited to secondary data. Further, other more rare outcomes (e.g., stillbirth) may be better suited to secondary designs where a higher sample size may be more achievable. Further guidance on the nuances of outcome selection across data sources would provide additional clarity to sponsors and aid in the streamlining and consistency across study protocols.

While this study observed only a small number of studies of vaccines, some differences between vaccine-associated and medicinal-associated outcomes were observed. These differences can likely be attributed to when the particular vaccines under study (e.g., RSVpreF) were recommended to be administered during pregnancy (mostly late in pregnancy which obviates the need to assess early-exposure outcomes such as spontaneous abortion). While many of the same outcomes are applicable to vaccine safety (e.g., stillbirth, preterm birth), some outcomes (e.g., Guillain-Barré Syndrome, anaphylaxis, myocarditis) may be more relevant to vaccine products. Existing guidance does not currently differentiate recommended outcomes by vaccine vs. medicinal exposures.

The FDA notes for pregnancy registries, “Determination of an adequate sample size depends on the objective(s) and design of the registry and the background rate of the outcome in the study population. If more than one pregnancy outcome is considered, sample size determination should be based on the outcome with the lowest background rate (e.g., MCM).” The EMA recommends using pooled data from multiple databases to maximize available sample size. However, neither regulatory agency provides an effect size that sponsors should target when evaluating particular outcomes. In this analysis, sample sizes from finalized studies were generally low, with a mean number of participants of 906.60 (median: 110). Target sample sizes were also low, with a mean of 504 (median: 360) participants (Table 1). Further, some studies were not able to recruit any exposed participants. To detect a risk ratio of at least 2.0 for major congenital malformations, the sample size for the exposed cohort would need to be approximately 600 (assuming 80% power, 1:1 matching between the exposed and unexposed, and a prevalence rate of 4% in the United States (United States Food and Drug Administration, 2002)). Of the studies that examined this outcome, only 6/13 (46.15%) of the finalized studies and 4/12 (33.33%) of the planned or ongoing studies met this sample size criteria. Similarly, a sample size of ∼1700 exposed individuals (assuming 80% power, 1:1 matching between the exposed and unexposed, and a prevalence rate of 0.5% in the United States (United States Food and Drug Administration, 2002)) is needed to detect a risk ratio of at least 3.0 for stillbirth. Of the studies that examined stillbirth, 4/11 (36.36%) finalized studies and none of the planned or ongoing studies met this sample size requirement. Of note, some studies did not state a singular target sample size and thus were excluded from this assessment.

Small sample sizes can sometimes be attributed to the medication or vaccine being uncommonly used in pregnancy, making it difficult to recruit enough patients. A systematic review of pregnancy registries for drugs and biologic products found the median enrollment of these studies to be 36 pregnancies (Bird et al., 2018). Another systematic review of these registries found that no registries were designed to have sufficient power to assess specific malformations (Gelperin et al., 2017). Indeed, there is great difficulty collecting enough data to significantly assess the risk of malformations. Thus, while sponsors may aim to capture a larger number of exposed individuals, actual capture may be much lower. This issue is evident primarily in primary data collection since secondary data use studies typically include more patients (Roque et al., 2022). In this analysis, the studies with larger sample sizes were typically secondary studies using existing databases and registries, therefore supporting previous findings. Additional guidance is needed from regulatory authorities on the effect sizes that should be targeted and whether the use of secondary vs. primary data collection impacts the targeted effect estimate.

Finally, both the FDA and EMA encourage sponsors to use existing disease-specific registries (across products) rather than develop product-specific registries. However, this recommendation is operationally difficult to implement for many reasons, including that studies are required or requested by regulatory agencies to evaluate different outcomes, making it difficult to join an existing registry. In this analysis, the mean number of outcomes evaluated was different across regulatory commitments (EMA-committed studies: 7 vs. FDA-committed studies: 12). Further, many differences across studies were identified even for studies committed to the same agency. Achieving harmonization on required outcomes is therefore critical to allow for the more efficient use of existing data.

## 5 Limitations

The following limitations should be considered when reviewing the conclusions from this work. First, the sample size of total studies evaluated was small, and subgroups of these studies were even smaller. There were only 30 applicable studies and therefore these studies may not be representative of the entire body of post-marketing pregnancy safety studies. Further there were only 4 FDA studies done before 2019 and 4 vaccine studies overall, which made it difficult to draw conclusions for some assessments. The small sample size of total studies may be caused by a few factors: (1) Not all studies in the HMA-EMA catalogue were included due to a lack of a publicly available protocol, which limited our review. It is possible that studies that included a publicly available protocol may have been different from studies that did not. However, a protocol was required to identify all the study outcomes for this analysis. (2) The studies included in this review relied on the built-in filters on the HMA-EMA catalogue. If a study measured pregnancy outcomes in the pregnant population but was not properly labeled as such in the catalogue, it may have been excluded based on the filters applied in Figure 1. It is possible that other technical issues within the site may have provided a smaller sample size than intended. (3) This review excluded data from spontaneous case reports which may have omitted several studies and their outcomes. This criterion was necessary to restrict to studies with pre-defined outcomes. Studies using spontaneous case report data often are designed to report any adverse outcome occurring in pregnancy and thus the outcomes that are summarized in the final report are determined by the outcomes observed in the reported cases, rather than a pre-defined outcome list. (4) Sponsors may have variations in their policies regarding whether a study needs to be registered in the HMA-EMA catalogue. While the EMA requires all post-authorization safety studies (PASS) to be registered, the FDA does not, and thus a large proportion of FDA-committed studies may be missing. Studies required by the two regulatory agencies are likely to differ substantially in design, data source, and selected outcomes. This limitation should therefore be considered when interpreting the results of this analysis.

Other limitations include the fact that some studies may have posted to the catalogue or updated their protocols or other relevant documents after the query date. An updated version of the protocol may capture additional outcomes that were not captured in this analysis.

Finally, to aid in interpretations, similar outcomes across studies were grouped together and summarized as a single outcome (see List A in the Supplementary Material for these categories). However, there was considerable variability in the specific outcomes captured within that broad category. For example, the outcome categorized as ‘infant growth and development’ included specific outcomes such as head circumference at birth and infant milestone status at 6 and 12 months of age. This variability within the outcomes summarized should be considered when interpreting the results of this analysis.

## 6 Conclusion

Overall, there is wide variation in the outcomes measured across existing and completed post-approval non-interventional safety studies in pregnant populations, which may be attributed to several factors, including regulatory agency of commitment, form of data collection, and product type. Despite a wide range of outcomes evaluated, few studies had sufficient sample size to thoroughly evaluate the risks for those outcomes. Additional guidance on specific outcomes to evaluate and at what effect size will ultimately (1) improve the efficiency in which sponsors can generate study protocols, begin analysis, and join existing registries; and (2) allow for easier comparison and interpretations of findings across studies.

## Data Availability

The raw data supporting the conclusions of this article will be made available by the authors, without undue reservation.

## References

[B1] Bird S. T. Gelperin K. Taylor L. Sahin L. Hammad H. Andrade S. E. (2018). Enrollment and retention in 34 United States pregnancy registries contrasted with the manufacturer’s capture of spontaneous reports for exposed pregnancies. Drug Saf. 41 (1), 87–94. 10.1007/s40264-017-0591-5 28840499 PMC8979755

[B2] European Medicines Agency (2005). Guideline on the exposure to medicinal products during pregnancy. Available online at: https://www.ema.europa.eu/en/documents/regulatory-procedural-guideline/guideline-exposure-medicinal-products-during-pregnancy-need-post-authorisation-data_en.pdf (Accessed July 22, 2025).

[B3] European Medicines Agency (2019). Guideline on good pharmacovigilance practices (GVP) - product- or Population-Specific Considerations III: pregnant and breastfeeding women. Available online at: https://www.ema.europa.eu/en/documents/scientific-guideline/draft-guideline-good-pharmacovigilance-practices-product-or-population-specific-considerations-iii-pregnant-and-breastfeeding-women_en.pdf (Accessed July 22, 2025).

[B4] Gelperin K. Hammad H. Leishear K. Bird S. T. Taylor L. Hampp C. (2017). A systematic review of pregnancy exposure registries: examination of protocol-specified pregnancy outcomes, target sample size, and comparator selection. Pharmacoepidemiol. Drug Saf. 26 (2), 208–214. 10.1002/pds.4150 28028914

[B5] Jones R. K. Upadhyay U. D. Weitz T. A. (2013). At what cost? Payment for abortion care by U.S. women. Women’s Health Issues 23 (3), e173–e178. 10.1016/j.whi.2013.03.001 23660430

[B6] MacDonald S. C. Guignard A. P. Munoz F. M. Dareng E. O. Davis K. J. de Avila Machado M. A. (2025). Post-approval safety studies of vaccines in pregnancy: available regulatory guidance and next steps towards the more efficient generation of safety evidence. Front. Drug Saf. Regul. 5, 1648854. 10.3389/fdsfr.2025.1648854 41383270 PMC12690392

[B7] Roque P. L. Durán C. E. Layton D. Poulentzas G. Lalagkas P. N. Kontogiorgis C. (2022). A landscape analysis of post-marketing studies registered in the EU PAS register and ClinicalTrials.gov focusing on pregnancy outcomes or breastfeeding effects: a contribution from the ConcePTION Project. Drug Safety 45 (4), 333–344. 10.1007/s40264-022-01154-7 35357659 PMC9021095

[B8] The European Network of Centres for Pharmacoepidemiology and Pharmacovigilance (ENCePP) (2025). Annex 2. Guidance on methods for the evaluation of medicines in pregnancy and breastfeeding - European Union. Available online at: https://encepp.europa.eu/encepp-toolkit/methodological-guide/annex-2-guidance-methods-evaluation-medicines-pregnancy-and-breastfeeding_en (Accessed July 22, 2025).

[B9] United States Food and Drug Administration (2002). Guidance for industry establishing pregnancy exposure registries. Available online at: https://www.fda.gov/media/75607/download (Accessed July 22, 2025).

[B10] United States Food and Drug Administration (2019). Postapproval pregnancy safety studies guidance for industry. Available online at: https://www.fda.gov/media/124746/download (Accessed July 22, 2025).

