# Supplementary figures and images for: Outcomes of post-approval non-interventional safety studies in pregnancy: wide variation demonstrates need for further standardization
Source: Front Drug Saf Regul. 2026 May 11;6:1805759. doi: 10.3389/fdsfr.2026.1805759 (PMC13198923; doi:10.3389/fdsfr.2026.1805759)

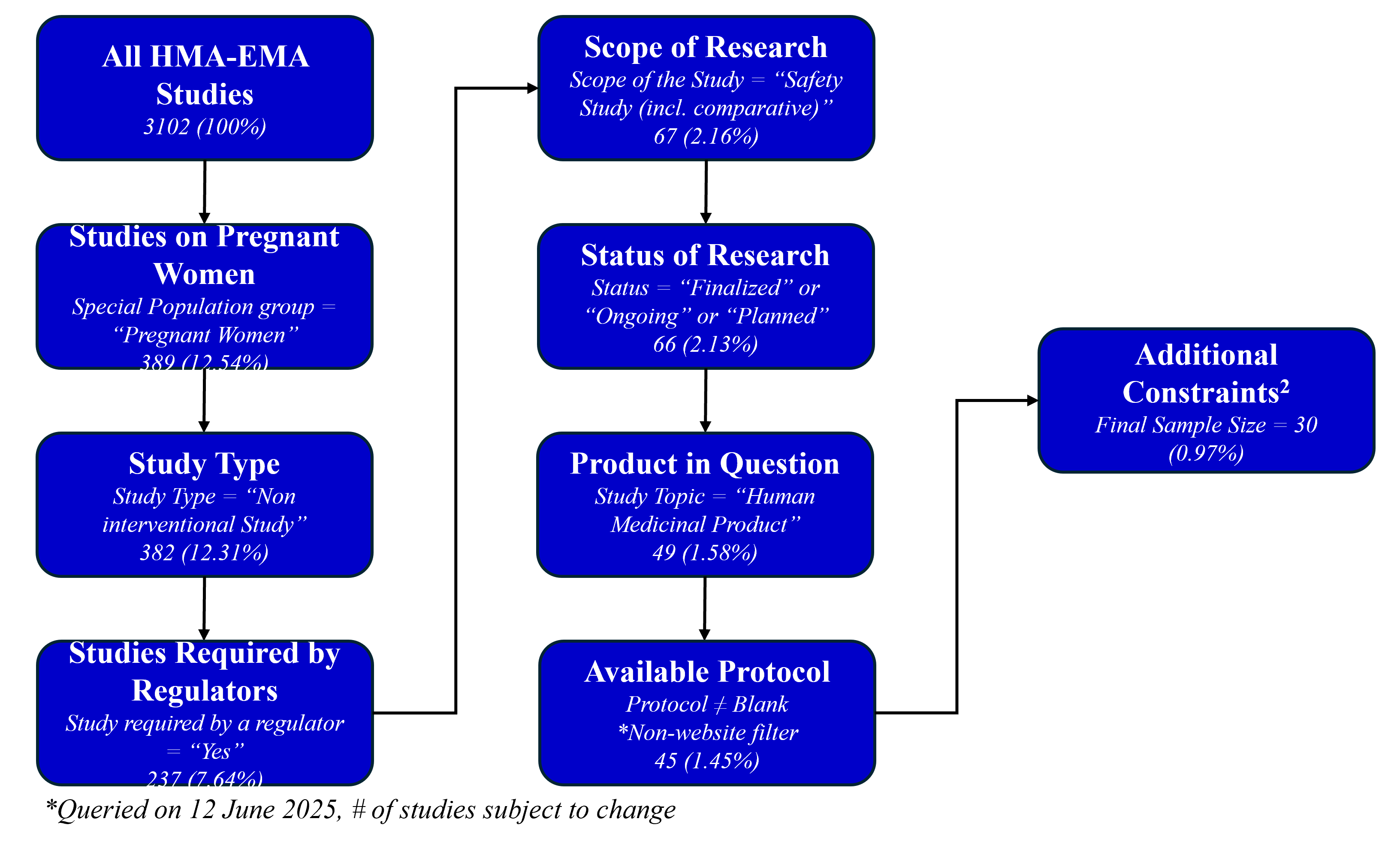

Supplement: Supplementary file 1 [file Presentation1.zip › Supplementary material presentation/fig1.tif]

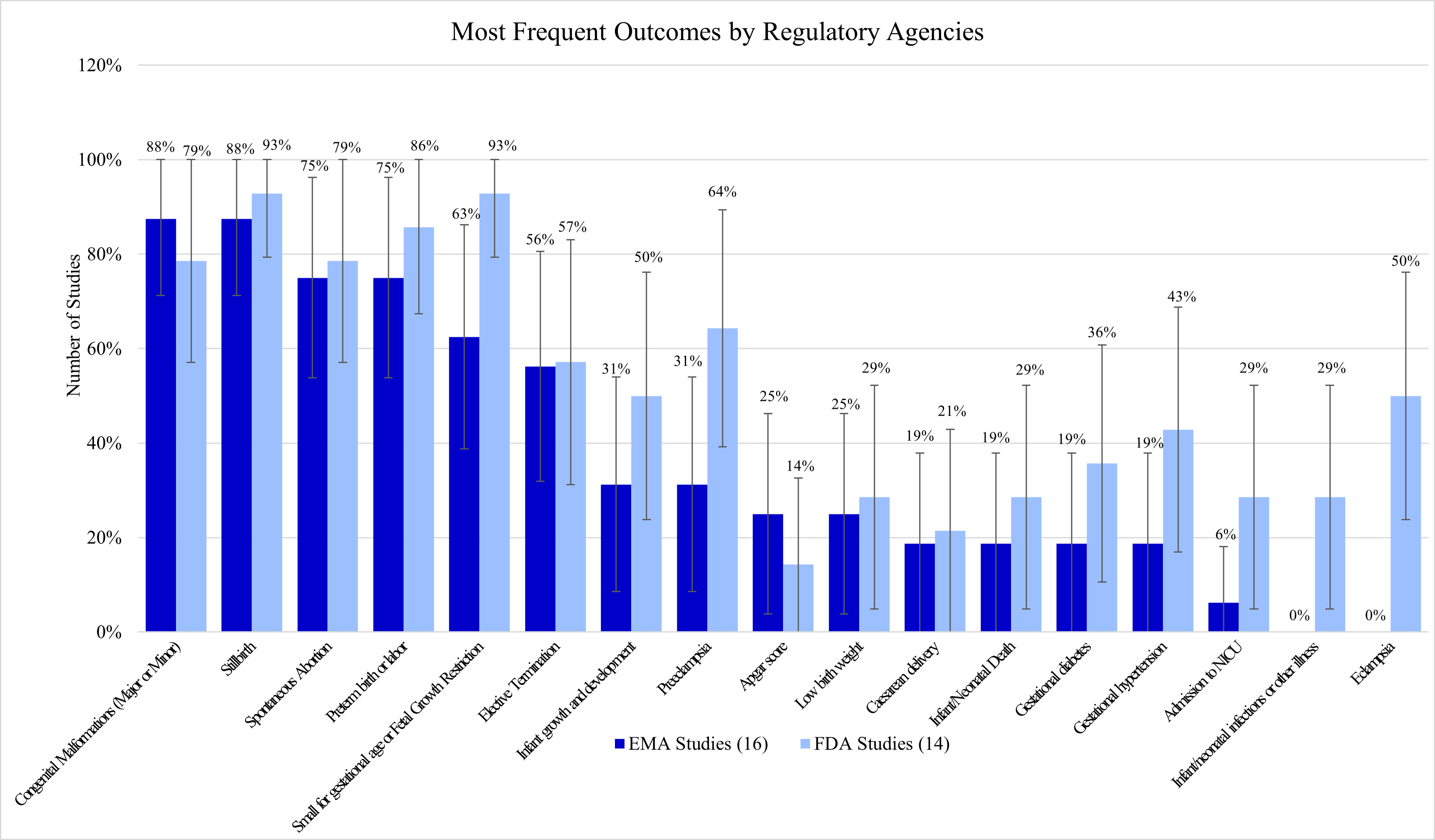

Supplement: Supplementary file 1 [file Presentation1.zip › Supplementary material presentation/fig2.tif]

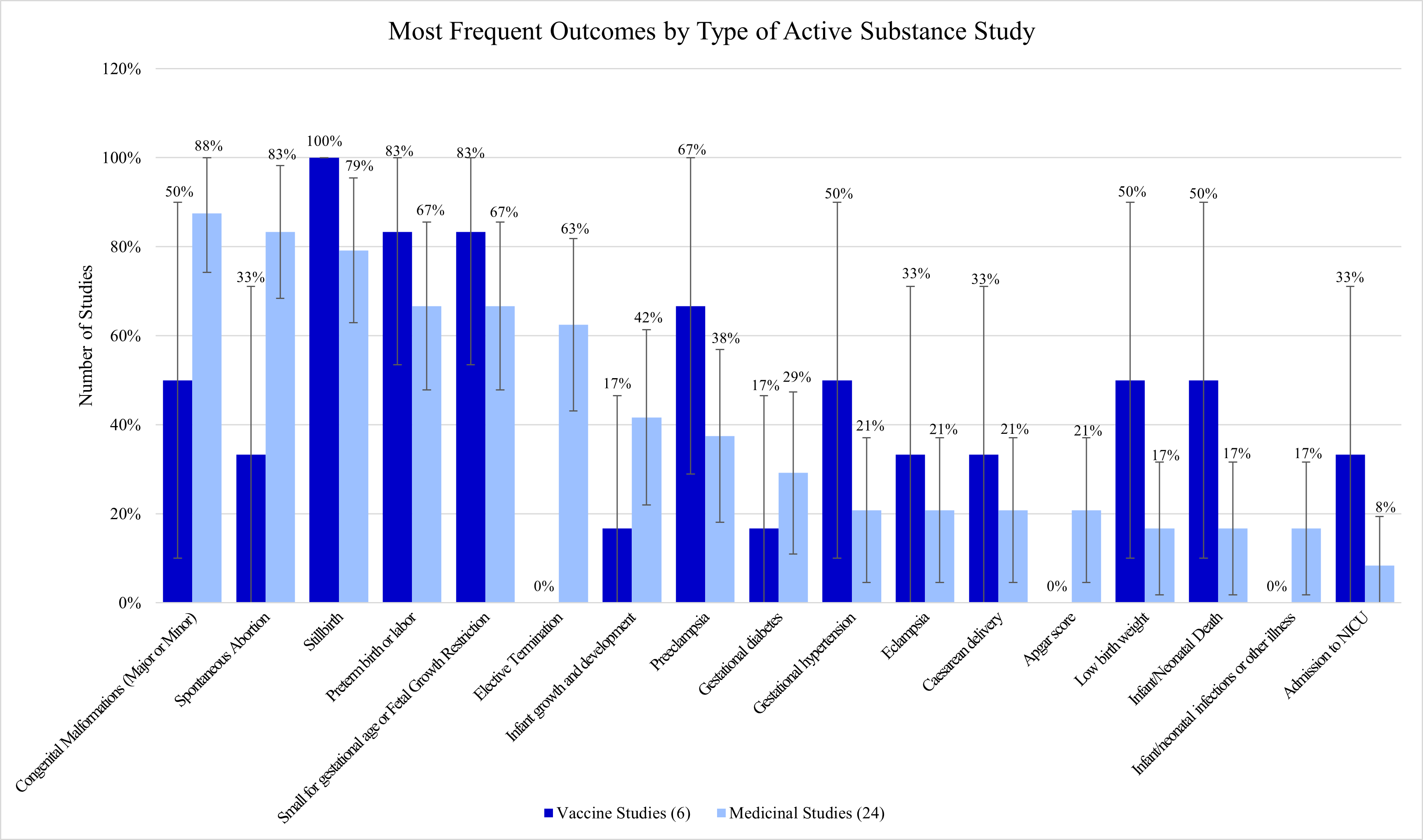

Supplement: Supplementary file 1 [file Presentation1.zip › Supplementary material presentation/fig3.tif]

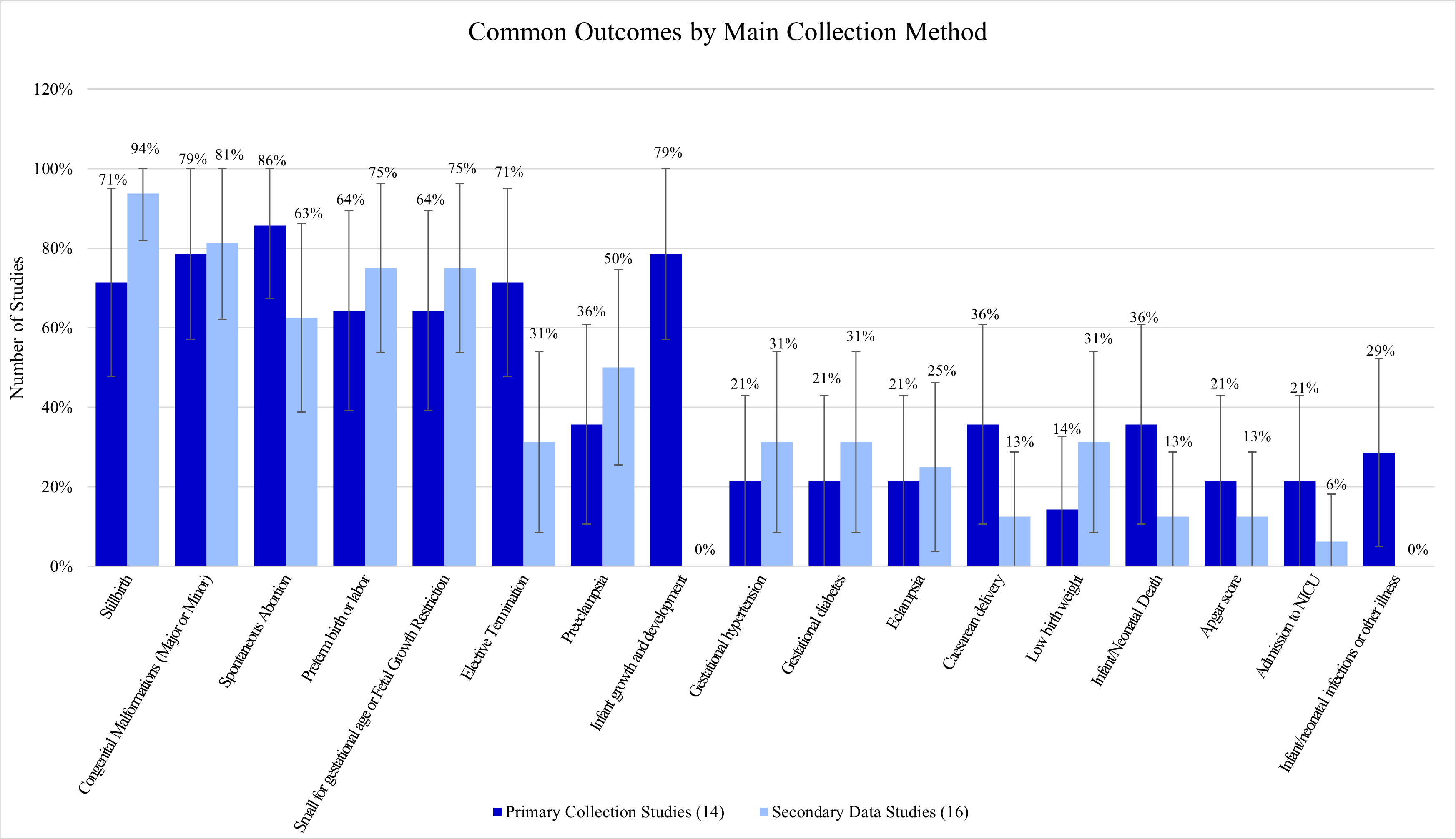

Supplement: Supplementary file 1 [file Presentation1.zip › Supplementary material presentation/fig4.tif]

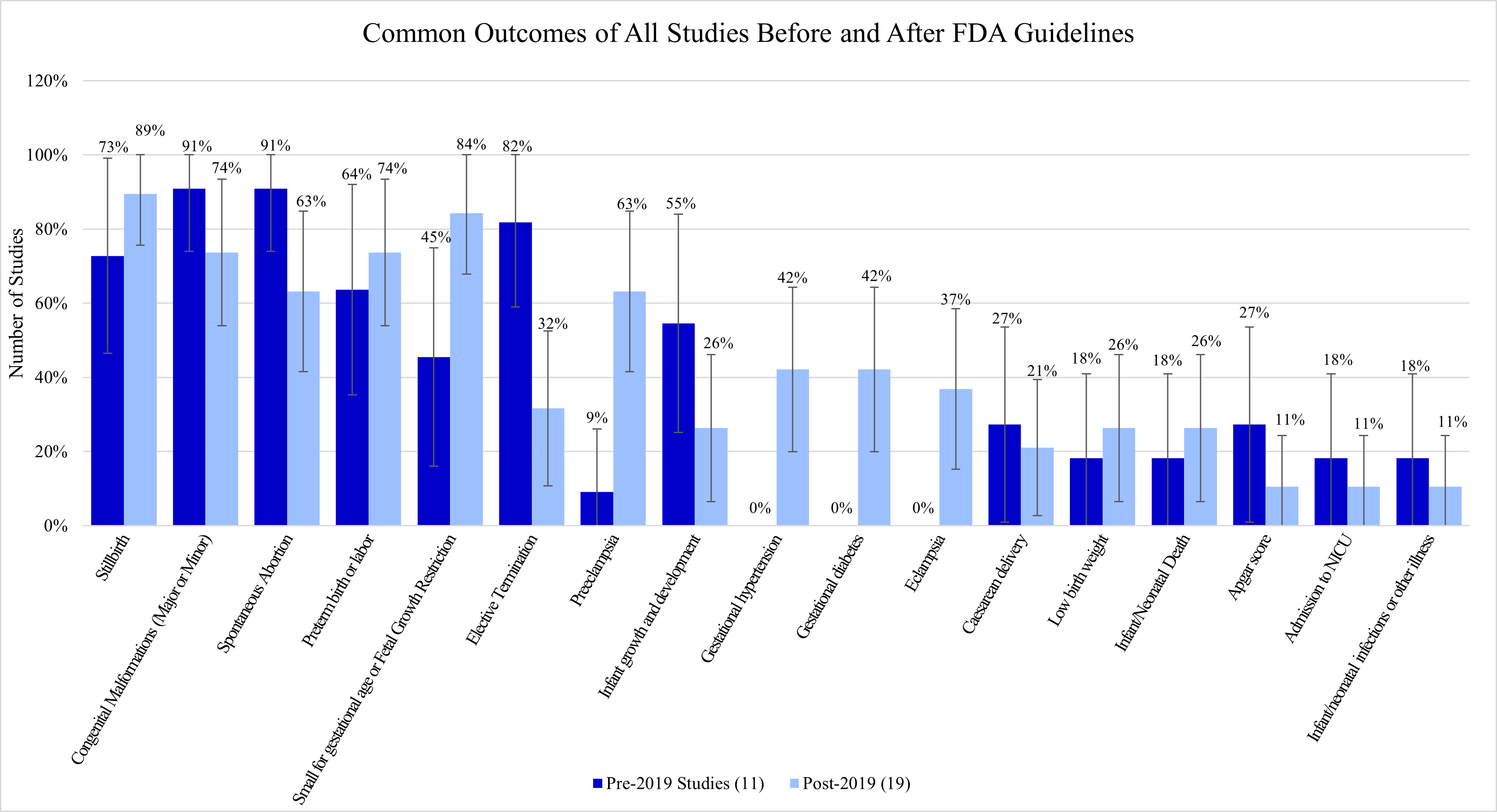

Supplement: Supplementary file 1 [file Presentation1.zip › Supplementary material presentation/fig5.tif]

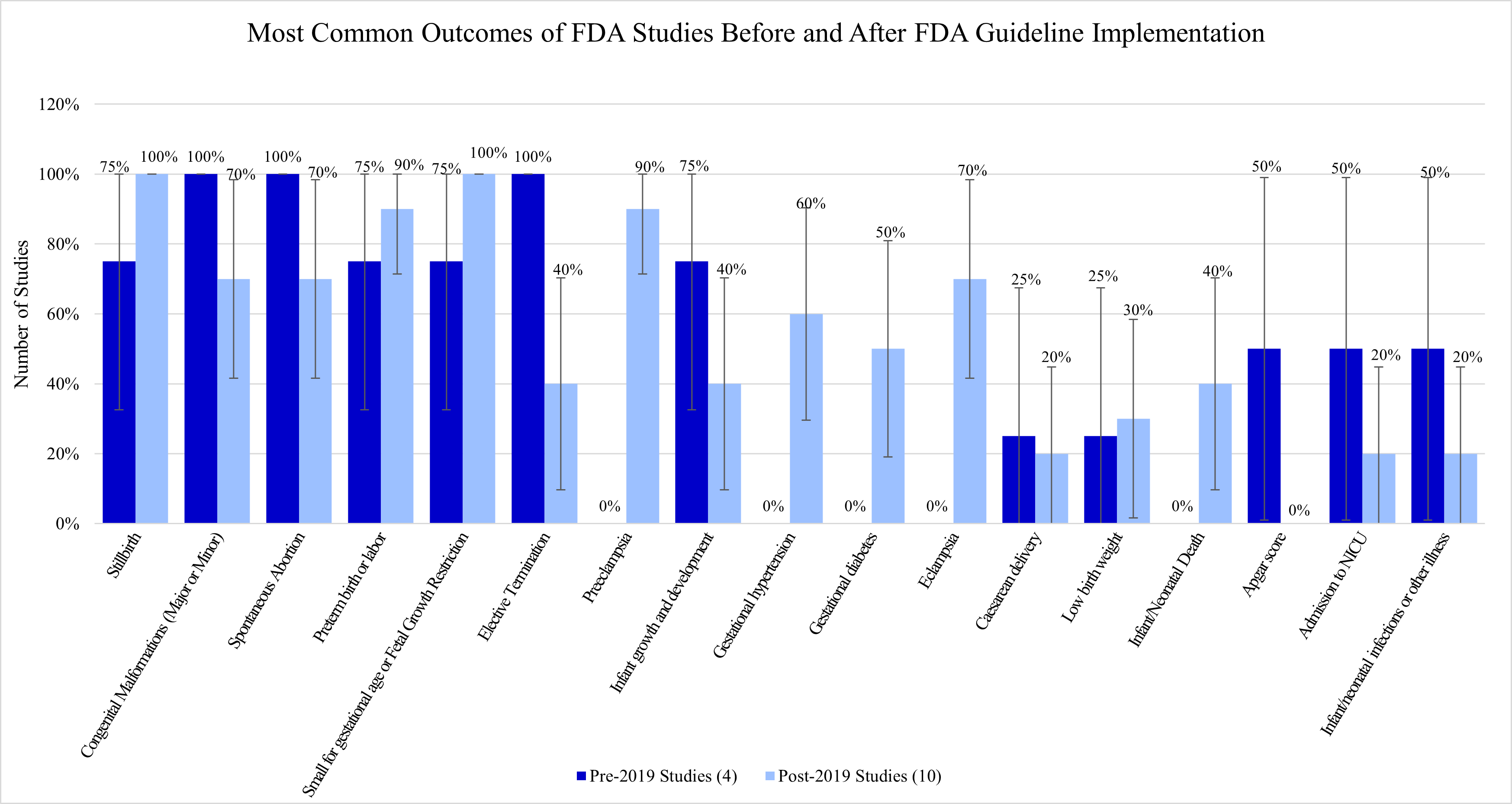

Supplement: Supplementary file 1 [file Presentation1.zip › Supplementary material presentation/fig6.tif]
