# Supplementary material for: Outcomes of post-approval non-interventional safety studies in pregnancy: wide variation demonstrates need for further standardization
Source: Front Drug Saf Regul. 2026 May 11;6:1805759. doi: 10.3389/fdsfr.2026.1805759 (PMC13198923; doi:10.3389/fdsfr.2026.1805759)
Supplement: Supplementary file 2 [file Table1.docx]

***Supplementary Material***

1. Supplementary Figures and Tables

| Supplementary Table 1. Consolidation of Outcomes^a b c^ | | |
| --- | --- | --- |
| Category of Outcome | Name of Outcome | Terms from Studies |
| Pregnancy Outcomes | Spontaneous abortion | Miscarriage  Spontaneous abortion  Spontaneous termination |
|  | Abortion complications | Abortion complications |
|  | Elective termination | Elective abortion  Elective termination  Induced abortion  Induced termination  Therapeutic abortion  Termination of pregnancy for fetal anomaly  Termination of pregnancy for fetal anomaly following prenatal diagnosis |
|  | Fetal loss, type not specified | Abortion  Fetal loss type not specified |
|  | Termination of pregnancy for fetal anomaly (TOPFA) | Termination of pregnancy for fetal anomaly  Termination of pregnancy for fetal anomaly following prenatal diagnosis |
|  | Ectopic pregnancy | Ectopic pregnancy  Ectopic |
|  | Molar pregnancy | Molar pregnancy |
|  | Stillbirth^c^ | Fetal death at 20 gestational weeks or later  Stillbirth |
|  | Preterm birth or labor | Premature birth  Premature delivery  Premature labor  Premature labor without preterm delivery  Preterm birth  Preterm delivery  Gestational age |
|  | Placental conditions | Placental abruption  Abnormal placenta |
|  | Premature rupture of membranes (PROM) | Premature rupture of membranes |
|  | Preterm PROM (PPROM) | Preterm premature rupture of membranes |
|  | Labor/delivery complications, not otherwise specified | Labor/delivery complications (eg, fetal distress, amniotic fluid abnormal) |
| Maternal Outcomes | Atrial fibrillation | Atrial fibrillation |
|  | Caesarean delivery | Caesarean delivery  Caesarean section  Mode of delivery |
|  | Preeclampsia | Preeclampsia |
|  | Eclampsia | Eclampsia |
|  | Gestational hypertension | Gestational hypertension |
|  | HELLP syndrome | (Hemolysis, elevated liver enzymes and low platelets) HELLP syndrome |
|  | Postpartum hypertension | Postpartum hypertension |
|  | Chronic hypertension superimposed with preeclampsia/eclampsia | Chronic hypertension superimposed with preeclampsia/eclampsia |
|  | Gestational diabetes | Gestational diabetes  Gestational diabetes mellitus |
|  | Major hypoglycemic events/metabolic control | Major hypoglycemic events  Metabolic control measured as HbA1c |
|  | Guillain-Barré syndrome | Guillain-Barré syndrome |
|  | Maternal death | Maternal death |
|  | Postpartum hemorrhage | Postpartum hemorrhage |
|  | Maternal length of stay | Prolonged maternal duration of hospital stay  Prolonged maternal length of stay |
|  | Thrombocytopenia | Thrombocytopenia |
|  | Polyneuropathies | Polyneuropathies |
|  | Unspecified maternal outcomes | Maternal medical problems during pregnancy |
|  | Immune-mediated demyelinating conditions | Acute disseminated encephalitis and encephalomyelitis  Acute transverse myelitis in demyelinating disease of central nervous system  Optic neuritis  Neuromyelitis optica  Other acute demyelinating diseases |
| Infant Outcomes | Admission to NICU | Admission to NICU  Admission in intensive care unit |
|  | Infant need for resuscitation | Need for resuscitation |
|  | Apgar score | Low Apgar score  Low Apgar score at 1 and 5 minutes  Low Apgar score at 5 minutes |
|  | Dysmaturity | Maturity assessment/dysmaturity |
|  | Congenital malformations (major or minor) | Congenital anomalies  Major congenital malformation  Minor congenital malformation  Recorded birth defect  Microcephaly  Malformation/anomalies diagnosed since initial report  Fetal malformations and neoplasms |
|  | Large for gestational age | Large for gestational age |
|  | Small for gestational age or fetal growth restriction | Fetal growth restriction  Small for gestational age  Alterations in fetal/infant growth  Fetal growth/development |
|  | Low birth weight | Low birth weight |
|  | Infant/neonatal death | Infant death  Neonatal death  Perinatal death  Death in newborn period |
|  | Infant/neonatal infections or other illness | Infant serious or opportunistic infections  Postnatal serious infections  Neonatal illness  Infant illness  Neonatal drug therapies  Infant drug therapies |
|  | Infant hospitalization | Infant hospitalization due to serious illness  Neonatal hospitalization  Infant hospitalization |
|  | Mechanical ventilation in neonatal period | Mechanical ventilation in neonatal period |
|  | Infant respiratory distress | Respiratory distress |
|  | Neurodevelopmental outcomes | Attention deficit hyperactivity disorder  Autism spectrum disorders  Learning disorders and intellectual disabilities  Learning disabilities  Adverse neurodevelopmental outcomes |
|  | Infant growth and development | Growth measurement and charts for the infant  Growth and health of infant  Infant postnatal growth deficiency  Infant milestone status at 6 and 12 months  Indications of delayed development  Infant developmental delay  Functional deficits observed during the first year of life in live born infants  Pervasive developmental disorders  Postnatal growth deficiency  Postnatal growth deficiency at 1 year of age  Development milestones  Alterations in fetal/infant growth  Weight  Length at birth  Head circumference at birth |
|  | Unspecified infant outcomes | Problems in neonatal period |
|  | Fetal Macrosomia | Fetal macrosomia |
| Other | Adverse events, unspecified | Adverse events |
| ^a^ Some study outcomes were excluded from this list if they were not an adverse safety outcome (e.g. livebirth), too product-specific (e.g. Infant response to IgG-tetanus antibody), or specific to breastfeeding populations  ^b^ These terms are the exact phrases found in the study protocols. This list is not a comprehensive list of alternative terms for each outcome.  ^c^ While intrauterine death is a common phrase used in studies, in this review it was only mentioned as a supporting definition for the outcome Stillbirth and therefore is not listed in this table. | | |

| **Supplementary Table 2. List of Ongoing and Planned Studies with Data Collected from Protocols** | | | | | |
| --- | --- | --- | --- | --- | --- |
| **Product (Medicine or Vaccine)** | **Protocol Year** | **Main Data Collection Method** | **Regulatory Commitment^a^** | **Target Exposed Sample Size** | **List of Outcomes**  **Number of Outcomes** |
| Evolocumab  (Medicine) (1) | 2019 | Primary | FDA | 75 | Spontaneous Abortion, Elective Termination, Stillbirth, Preterm birth or labor, Congenital Malformations (Major or Minor), Small for Gestational Age or Fetal Growth Restriction, Infant growth and development, Infant/neonatal infection or other illness, Neurodevelopmental outcomes  N=9 |
| Burosumab (2) | 2021 | Primary | EMA | 400 | Infant Growth and Development, Apgar Score, Caesarean Delivery  N=2 |
| Rimegepant (Medicine) (3) | 2022 | Secondary | FDA | 1152 | Spontaneous Abortion, Stillbirth, Elective Termination, Preterm birth or labor, Congenital Malformations (Major or Minor), Small for Gestational Age or Fetal growth restriction, Preeclampsia, Eclampsia  N=8 |
| Paxlovid (Medicine) (4) | 2023 | Secondary | EMA and MHRA | *No single size provided | Spontaneous Abortion, Elective Termination, Stillbirth, Preterm birth or labor, Congenital Malformations (Major or Minor), Small for Gestational Age or Fetal growth restriction, Gestational Diabetes, Gestational Hypertension, Postpartum hemorrhage, Maternal death  N=10 |
| Tezepelumab (Medicine) (5) | 2023 | Secondary | EMA | 190* with 1:2 matching ratio | Elective Termination, Termination of Pregnancy for Fetal Anomaly (TOPFA), Stillbirth, Spontaneous Abortion, Ectopic Pregnancy, Preterm birth or labor, Congenital Malformations (Major or Minor), Small for Gestational Age or Fetal Growth Restriction, Low Birth Weight, Preeclampsia, Caesarean delivery  N=11 |
| Ublituximab  (Medicine) (6) | 2024 | Secondary | FDA | 540 | Spontaneous Abortion, Stillbirth, Preterm birth or labor, Placental Conditions, Congenital Malformations (Major or Minor), Small for Gestational Age or Fetal Growth Restriction, Eclampsia, Preeclampsia, Gestational Diabetes  N=9 |
| Zavegepant  (Medicine) (7) | 2024 | Primary | FDA | 364 | Spontaneous Abortion, Elective Termination, Stillbirth, Congenital Malformations (Major or Minor), Small for Gestational Age or Fetal Growth Restriction, Infant growth and development, Preeclampsia, Eclampsia, Gestational Diabetes, Gestational Hypertension  N=10 |
| Omaveloxolone (Medicine) (8) | 2024 | Primary | FDA | 10-20 | Elective Termination, Spontaneous Abortion, Fetal loss (type not specified), Preterm birth or labor, Stillbirth, Congenital Malformations (Major or Minor), Small for Gestational Age or Fetal Growth Restriction, Infant/Neonatal death, Infant growth and development, Infant hospitalization, Infant/neonatal infection or other illness, Gestational Diabetes, Preeclampsia  N=13 |
| Respiratory syncytial virus vaccine (bivalent, recombinant) (Vaccine) (9) | 2024 | Secondary | EMA and FDA | 600 | Stillbirth, Preterm birth or labor, Low Birth Weight, Small for Gestational age or Fetal Growth Restriction, Gestational Hypertension, Preeclampsia, Guillain-Barré Syndrome  N=7 |
| Bivalent respiratory syncytial virus (RSV) stabilized prefusion F subunit vaccine (RSVpreF) (Vaccine) (10) | 2024 | Secondary | FDA | 1031 | Preterm birth or labor, Stillbirth, Premature rupture of membranes (PROM), Preterm PROM (PPROM), Small for Gestational Age or Fetal Growth Restriction, Large for Gestational Age, Low Birth Weight, Admission to NICU, Mechanical ventilation in neonatal period, Infant/Neonatal death, Infant growth and development, Preeclampsia, Eclampsia, Gestational Hypertension, HELLP syndrome, Postpartum hypertension, Chronic hypertension superimposed with preeclampsia/eclampsia, Thrombocytopenia, Guillain-Barré syndrome, Polyneuropathies, Atrial fibrillation, Maternal death, Caesarean delivery, Maternal length of stay, Immune-mediated demyelinating conditions  N=25 |
| BIMERVAX® emulsion for injection  COVID-19 vaccine, recombinant, adjuvanted (Vaccine) (11) | 2024 | Secondary | EMA | *No single size provided | Spontaneous Abortion, Stillbirth, Preterm birth or labor, Small for Gestational Age or Fetal Growth Restriction, Congenital Malformations (Major or Minor), Infant/Neonatal death, Gestational Diabetes, Preeclampsia, Maternal death  N=9 |
| Baricitinib (Medicine) (12) | 2025 | Secondary | FDA | 434 | Spontaneous Abortion, Stillbirth, Preterm birth or labor, Congenital Malformations (Major or Minor), Small for Gestational Age or Fetal Growth Restriction  N=5 |
| Zavegepant (Medicine) (13) | 2025 | Secondary | FDA | 884 | Spontaneous Abortion, Stillbirth, Preterm birth or labor, Congenital Malformations (Major or Minor), Small for Gestational Age or Fetal Growth Restriction, Preeclampsia, Eclampsia, Gestational Diabetes, Gestational Hypertension  N=9 |
| Ritlecitinib (Medicine) (14) | 2025 | Primary | FDA | 364 | Spontaneous Abortion, Elective Termination, Stillbirth, Congenital Malformations (Major or Minor), Small for Gestational Age or Fetal Growth Restriction, Infant growth and development, Preeclampsia, Eclampsia  N=14 |
| Bivalent respiratory syncytial virus (RSV)  stabilized prefusion F subunit vaccine  (RSVpreF) (15) (Vaccine) | 2025 | Secondary | FDA | *No single size provided | Stillbirth, Preterm birth or labor, Premature rupture of membranes (PROM), Preterm PROM (PPROM), Small for Gestational Age or Fetal Growth Restriction, Large for Gestational Age, Low Birth Weight, Admission to NICU, Mechanical ventilation in neonatal period, Infant/Neonatal death, Thrombocytopenia, Guillain-Barré syndrome, Polyneuropathies, Atrial fibrillation, Caesarean delivery, Maternal length of stay, Gestational hypertension, Preeclampsia, Eclampsia, HELLP syndrome, Chronic hypertension superimposed with preeclampsia/eclampsia, Postpartum hypertension, Immune-mediated demyelinating conditions  N=23 |
| Abbreviations: EMA = Heads of Medicines Agencies-European Medicine Agency, FDA = Food and Drug Administration  ^a^ These commitments were explicitly stated in the protocol though the study may be committed to other regulatory agencies | | | | | |

| **Supplementary Table 3. List of Finalized Studies with Data Collected from Protocols** | | | | | | |
| --- | --- | --- | --- | --- | --- | --- |
| **Product (Medicine or Vaccine)** | **Year of Final**  **Protocol** | **Data Collection Methods** | **Regulatory Commitment^a^** | **Target Exposed Sample Size** | **Final Exposed Sample Size** | **List of Outcomes**  **Number of Studies** |
| Ivacaftor (Medicine) (16) | 2013 | Secondary | EMA | *does not specify | UK: 158  US: 484 | Stillbirth, Spontaneous Abortion, Elective Termination, Preterm birth or labor, Congenital Malformations (Major or Minor)  N=5 |
| Human normal immunoglobulin  (Medicine) (17) | 2015 | Secondary | FDA | No prespecified minimum sample size | 9 | Spontaneous Abortion, Elective Termination, Ectopic Pregnancy, Molar Pregnancy, Placental Conditions, Labor/delivery complications (not otherwise specified), Small for Gestational Age or Fetal Growth Restriction, Low Birth Weight, Congenital Malformations (Major or Minor), Dysmaturity, Apgar Score, Infant growth and development, Admission to NICU, Infant need for resuscitation, Infant/neonatal infections or other illness, Infant hospitalization, Caesarean Delivery  N=17 |
| Umeclidinium (Medicine) (18) | 2015 | Primary | Japan Pharmaceutical Affairs Law and Good Post-marketing Study Practice | 1000 (total pts not just pregnant cohort) | 0 | Spontaneous Abortion, Fetal loss (type not specified), Adverse events (unspecified), Caesarean Delivery  N=4 |
| Alemtuzumab (Medicine) (19) | 2016 | Primary | EMA | 204 | 42 | Spontaneous Abortion, Stillbirth, Elective Termination, Preterm birth or labor, Congenital Malformations (Major or Minor), Small for Gestational Age or Fetal Growth Restriction, Infant growth and development  N=7 |
| Insulin detemir (Medicine) (20) | 2017 | Primary | EMA | 1222 | 764 | Preterm birth or labor, Spontaneous Abortion, Elective Termination, Infant/Neonatal death, Congenital Malformation (Major or Minor), Infant growth and development, Fetal Macrosomia, Major hypoglycemic events/metabolic control, Preeclampsia  N=9 |
| Vedolizumab (Medicine) (21) | 2017 | Primary | FDA and EMA | 2500 (total pts not just pregnant cohort) | 100 | Preterm birth or labor, Stillbirth, Spontaneous Abortion, Elective Abortion, Apgar Score, Infant Respiratory Distress, Admissions to NICU, Congenital Malformations (Major or Minor)  N=8 |
| Glycerol phenylbutyrate (22) | 2018 | Primary | EMA | 200 (total pts not just pregnant cohort) | 0 | Spontaneous Abortion, Stillbirth, Elective Termination, Congenital Malformations (Major or Minor), Unspecified Infant Outcomes, Infant/Neonatal death, Infant Growth and Development, Caesarean Delivery, Unspecified Maternal Outcomes  N=9 |
| Pregabalin (Medicine)^1^ (23) | 2018 | Secondary | EMA | ≥1000 | Denmark: 332  Finland: 995*  Norway: 317*  Sweden: 1275 | Stillbirth, Preterm birth or labor, Congenital Malformations (Major or Minor), Low Birth Weight, Small for Gestational Age or Fetal Growth Restriction, Apgar Score, Neurodevelopmental Outcomes  N=7 |
| Teriflunomide (Medicine) (24) | 2018 | Primary | FDA and EMA | 196 | 43 | Spontaneous Abortion, Elective Termination, Termination of Pregnancy for Fetal Anomaly (TOPFA), Ectopic pregnancy, Preterm birth, Molar Pregnancy, Stillbirth, Congenital Malformations (Major or Minor), Small for Gestational Age or Fetal Growth Restriction, Infant growth and development  N=10 |
| Tezacaftor and Ivacaftor (Medicine) (25) | 2019 | Secondary | EMA | *does not specify | US: 2245  Germany: 318  UK: 689 | Stillbirth, Spontaneous Abortion, Elective Termination, Congenital Malformations (Major or Minor)  N=4 |
| GARDASIL (Vaccine) (26) | 2020 | Secondary | CDE | Redacted | 168 | Stillbirth, Congenital Malformations (Major or Minor)  N=2 |
| Gabapentin (Medicine)^2^ (27) | 2020 | Secondary | EMA | 1743 | Denmark: 472*  Finland: 261*  Norway: 496*  Sweden: 806 | Stillbirth, Preterm birth or labor, Congenital Malformations (Major or Minor), Low Birth Weight, Small for Gestational Age or Fetal Growth Restriction, Apgar Score, Neurodevelopmental Outcomes  N=7 |
| Misoprostol, Diclofenac + Misoprostol  (Medicine) (28) | 2020 | Secondary | Agenzia Italiana del Farmaco | 78-300 | 110 | Abortion complications  N=1 |
| AZD1222 (Vaccine)^3^ (29) | 2021 | Primary | EMA | 15000 (total pts not just pregnant cohort) | 0 | Spontaneous Abortion, Stillbirth, Preterm birth or labor, Congenital Malformations (Major or Minor), Small for Gestational Age or Fetal Growth Restriction  N=5 |
| Tacrolimus (Medicine) (30) | 2021 | Secondary | EMA | 850 (total from initial feasibility assessments) | As of Dec 2020: 3515 | Spontaneous Abortion, Stillbirth, Congenital Malformations (Major or Minor), Small for Gestational Age or Fetal Growth Restriction, Preeclampsia, Gestational Hypertension, Gestational Diabetes  N=7 |
| Abbreviations: EMA = Heads of Medicines Agencies-European Medicine Agency, FDA = Food and Drug Administration, CDE = Center for Drug Evaluation  ^a^ These commitments were explicitly stated in the protocol. The study may be committed to other regulatory agencies.  ^1^ Countries marked with an asterisk rounded individuals in some cohorts to the nearest 5^th^ to avoid identification of the individual, therefore final sample size count may not be fully accurate  ^2^ Countries marked with an asterisk rounded individuals and thus the final sample size may not be fully accurate  ^3^ Study was closed early due to enrollment challenges, instead there were subsequent studies done with a different design | | | | | | |

| **Supplementary Table 4. Percentage of Regulatory Agency Commitments^a^ by Most Common Outcome** | | | | |
| --- | --- | --- | --- | --- |
| **Common Outcomes**^b^  Total Number of Studies^c^ | **EMA Committed Studies**  **N=16** | **95% CI** | **FDA Committed Studies**  **N=14** | **95% CI** |
|  | **n (%)** |  | **n (%)** |  |
| Stillbirth  N=25 | 14 (87.50%) | [71.29%, 100.00%] | 13 (92.86%) | [79.37%, 100.00%] |
| Congenital malformations (major or minor)  N=24 | 14 (87.50%) | [71.29%, 100.00%] | 11 (78.57%) | [57.08%, 100.00%] |
| Spontaneous abortion  N=22 | 12 (75.00%) | [53.78%, 96.22%] | 11 (78.57%) | [57.08%, 100.00%] |
| Preterm birth or labor  N=21 | 12 (75.00%) | [53.78%, 96.22%] | 12 (85.71%) | [67.38%, 100.00%] |
| Small for gestational age or fetal growth restriction  N=21 | 10 (62.50%) | [38.78%, 86.22%] | 13 (92.86%) | [79.37%, 100.00%] |
| Elective termination  N=15 | 9 (56.25%) | [31.94%, 80.56%] | 8 (57.14%) | [31.22%, 83.06%] |
| Preeclampsia  N=13 | 5 (31.25%) | [8.54%, 53.96%] | 9 (64.29%) | [39.19%, 89.39%] |
| Infant growth and development  N=11 | 5 (31.25%) | [8.54%, 53.96%] | 7 (50.00%) | [23.81%, 76.19%] |
| Gestational hypertension  N=8 | 3 (18.75%) | [0.00%, 37.88%] | 6 (42.86%) | [16.94%, 68.78%] |
| Gestational diabetes  N=8 | 3 (18.75%) | [0.00%, 37.88%] | 5 (35.71%) | [10.61%, 60.81%] |
| Eclampsia  N=7 | 0 (0.00%) | [0.00%, 0.00%] | 7 (50.00%) | [23.81%, 76.19%] |
| Caesarean delivery  N=7 | 3 (18.75%) | [0.00%, 37.88%] | 3 (21.43%) | [0.00%, 42.92%] |
| Low birth weight  N=7 | 4 (25.00%) | [3.78%, 46.22%] | 4 (28.57%) | [4.91%, 52.23%] |
| Infant/neonatal Death  N=7 | 3 (18.75%) | [0.00%, 37.88%] | 4 (28.57%) | [4.91%, 52.23%] |
| Apgar score  N=5 | 4 (25.00%) | [3.78%, 46.22%] | 2 (14.29%) | [0.00%, 32.62%] |
| Admission to NICU  N=4 | 1 (6.25%) | [0.00%, 18.11%] | 4 (28.57%) | [4.91%, 52.23%] |
| Infant/neonatal infections or other illness  N=4 | 0 (0.00%) | [0.00%, 0.00%] | 4 (28.57%) | [4.91%, 52.23%] |
| Abbreviations: EMA = European Medicine Agency; FDA = Food and Drug Administration  ^a^ There were 4 studies that were committed to an agency other than the EMA or FDA and were excluded from the table.  ^b^ Only outcomes that were captured in at least 4 studies are listed.  ^c^ Percentages across the rows may not total 100% since there were studies that were committed to both EMA and FDA, therefore counted in both columns. | | | | |

| **Supplementary Table 5. Percentage of Outcomes Measured by Type of Active Substance Study** | | | | |
| --- | --- | --- | --- | --- |
| **Common Outcomes^a^**  Total Number of Studies | **Vaccine Studies**  **N=6** | **95% CI** | **Medicine Studies**  **N=24** | **95% CI** |
|  | **n (%)** |  | **n (%)** |  |
| Stillbirth  N=25 | 6 (100.00%) | [100.00%, 100.00%] | 19 (79.17%) | [62.92%, 95.42%] |
| Congenital malformations (major or minor)  N=24 | 3 (50.00%) | [9.99%, 90.01%] | 21 (87.5%) | [74.27%, 100.00%] |
| Spontaneous abortion  N=22 | 2 (33.33%) | [0.00%, 71.05%] | 20 (83.33%) | [68.42%, 98.24%] |
| Preterm birth or labor  N=21 | 5 (83.33%) | [53.51%, 100.00%] | 16 (66.67%) | [47.81%, 85.53%] |
| Small for gestational age or fetal growth restriction  N=21 | 5 (83.33%) | [53.51%, 100.00%] | 16 (66.67%) | [47.81%, 85.53%] |
| Elective termination  N=15 | 0 (0.00%) | [0.00%, 0.00%] | 15 (62.5%) | [43.13%, 81.87%] |
| Preeclampsia  N=13 | 4 (66.67%) | [28.95%, 100.00%] | 9 (37.5%) | [18.13%, 56.87%] |
| Infant growth and development  N=11 | 1 (16.67%) | [0.00%, 46.49%] | 10 (41.67%) | [21.95%, 61.39%] |
| Gestational hypertension  N=8 | 3 (50.00%) | [9.99%, 90.01%] | 5 (20.83%) | [4.58%, 37.08%] |
| Gestational diabetes  N=8 | 1 (16.67%) | [0.00%, 46.49%] | 7 (29.17%) | [10.98%, 47.36%] |
| Eclampsia  N=7 | 2 (33.33%) | [0.00%, 71.05%] | 5 (20.83%) | [4.58%, 37.08%] |
| Caesarean delivery  N=7 | 2 (33.33%) | [0.00%, 71.05%] | 5 (20.83%) | [4.58%, 37.08%] |
| Low birth weight  N=7 | 3 (50.00%) | [9.99%, 90.01%] | 4 (16.67%) | [1.76%, 31.58%] |
| Infant/neonatal Death  N=7 | 3 (50.00%) | [9.99%, 90.01%] | 4 (16.67%) | [1.76%, 31.58%] |
| Apgar score  N=5 | 0 (0.00%) | [0.00%, 0.00%] | 5 (20.83%) | [4.58%, 37.08%] |
| Admission to NICU  N=4 | 2 (33.33%) | [0.00%, 71.05%] | 2 (8.33%) | [0.00%, 19.39%] |
| Infant/neonatal infections or other illness  N=4 | 0 (0.00%) | [0.00%, 0.00%] | 4 (16.67%) | [1.76%, 31.58%] |
| ^a^ Only outcomes that were captured in at least 4 studies are listed | | | | |

| **Supplementary Table 6. Percentage of Outcomes by Main Data Collection Method** | | | | |
| --- | --- | --- | --- | --- |
| **Common Outcomes^a^**  Total Number of Studies | **Primary**  **N=14** | **95% CI** | **Secondary**  **N=16** | **95% CI** |
|  | **n (%)** |  | **n (%)** |  |
| Stillbirth  N=25 | 10 (71.43%) | [47.76%, 95.09%] | 15 (93.75%) | [81.89%, 100.00%] |
| Congenital malformations (major or minor)  N=24 | 11 (78.57%) | [57.08%, 100.00%] | 13 (81.25%) | [62.12%, 100.00%] |
| Spontaneous abortion  N=22 | 12 (85.71%) | [67.38%, 100.00%] | 10 (62.5%) | [38.78%, 86.22%] |
| Preterm birth or labor  N=21 | 9 (64.29%) | [39.19%, 89.39%] | 12 (75.00%) | [53.78%, 96.22%] |
| Small for gestational age or fetal growth restriction  N=21 | 9 (64.29%) | [39.19%, 89.39%] | 12 (75.00%) | [53.78%, 96.22%] |
| Elective termination  N=15 | 10 (71.43%) | [47.76%, 95.09%] | 5 (31.25%) | [8.54%, 53.96%] |
| Preeclampsia  N=13 | 5 (35.71%) | [10.61%, 60.81%] | 8 (50%) | [25.50%, 74.50%] |
| Infant growth and development  N=11 | 11 (78.57%) | [57.08%, 100.00%] | 0 (0.00%) | [0.00%, 0.00%] |
| Gestational hypertension  N=8 | 3 (21.43%) | [0.00%, 42.92%] | 5 (31.25%) | [8.54%, 53.96%] |
| Gestational diabetes  N=8 | 3 (21.43%) | [0.00%, 42.92%] | 5 (31.25%) | [8.54%, 53.96%] |
| Eclampsia  N=7 | 3 (21.43%) | [0.00%, 42.92%] | 4 (25.00%) | [3.78%, 46.22%] |
| Caesarean delivery  N=7 | 5 (35.71%) | [10.61%, 60.81%] | 2 (12.50%) | [0.00%, 28.71%] |
| Low birth weight  N=7 | 2 (14.29%) | [0.00%, 32.62%] | 5 (31.25%) | [8.54%, 53.96%] |
| Infant/neonatal Death  N=7 | 5 (35.71%) | [10.61%, 60.81%] | 2 (12.50%) | [0.00%, 28.71%] |
| Apgar score  N=5 | 3 (21.43%) | [0.00%, 42.92%] | 2 (12.50%) | [0.00%, 28.71%] |
| Admission to NICU  N=4 | 3 (21.43%) | [0.00%, 42.92%] | 1 (6.25%) | [0.00%, 18.11%] |
| Infant/neonatal infections or other illness  N=4 | 4 (28.57%) | [4.91%, 52.24%] | 0 (0.00%) | [0.00%, 0.00%] |
| ^a^ Only outcomes that were captured in at least 4 studies are listed | | | | |

| **Supplementary Table 7. Percentage of All Studies with a Protocol Year Dated^a^ Before and After FDA Guideline Implementation by Most Common Outcomes** | | | | |
| --- | --- | --- | --- | --- |
| **Common Outcomes^b^**  Total Number of Studies | **Pre-2019**  **N=11** | **95% CI** | **Post-2019**  **N=19** | **95% CI** |
|  | **n (%)** |  | **n (%)** |  |
| Stillbirth  N=25 | 8 (72.73%) | [46.41%, 99.05%] | 17 (89.47%) | [75.67%, 100.00%] |
| Congenital malformations (major or minor)  N=24 | 10 (90.91%) | [73.92%, 100.00%] | 14 (73.68%) | [53.88%, 93.48%] |
| Spontaneous abortion  N=22 | 10 (90.91%) | [73.92%, 100.00%] | 12 (63.16%) | [41.47%, 84.85%] |
| Preterm birth or labor  N=21 | 7 (63.64%) | [35.21%, 92.06%] | 14 (73.68%) | [53.88%, 93.48%] |
| Small for gestational age or fetal growth restriction  N=21 | 5 (45.45%) | [16.03%, 74.88%] | 16 (84.21%) | [67.81%, 100.00%] |
| Elective termination  N=15 | 9 (81.82%) | [59.03%, 100.00%] | 6 (31.58%) | [10.68%, 52.48%] |
| Preeclampsia  N=13 | 1 (9.09%) | [0.00%, 26.08%] | 12 (63.16%) | [41.47%, 84.85%] |
| Infant growth and development  N=11 | 6 (54.55%) | [25.12%, 83.97%] | 5 (26.32%) | [6.52%, 46.12%] |
| Gestational hypertension  N=8 | 0 (0.00%) | [0.00%, 0.00%] | 8 (42.11%) | [19.90%, 64.31%] |
| Gestational diabetes  N=8 | 0 (0.00%) | [0.00%, 0.00%] | 8 (42.11%) | [19.90%, 64.31%] |
| Eclampsia  N=7 | 0 (0.00%) | [0.00%, 0.00%] | 7 (36.84%) | [15.15%, 58.53%] |
| Caesarean delivery  N=7 | 3 (27.27%) | [0.95%, 53.59%] | 4 (21.05%) | [2.72%, 39.38%] |
| Low birth weight  N=7 | 2 (18.18%) | [0.00%, 40.97%] | 5 (26.32%) | [6.52%, 46.12%] |
| Infant/neonatal Death  N=7 | 2 (18.18%) | [0.00%, 40.97%] | 5 (26.32%) | [6.52%, 46.12%] |
| Apgar score  N=5 | 3 (27.27%) | [0.95%, 53.59%] | 2 (10.53%) | [0.00%, 24.33%] |
| Admission to NICU  N=4 | 2 (18.18%) | [0.00%, 40.97%] | 2 (10.53%) | [0.00%, 24.33%] |
| Infant/neonatal infections or other illness  N=4 | 2 (18.18%) | [0.00%, 40.97%] | 2 (10.53%) | [0.00%, 24.33%] |
| Abbreviations: FDA = Food and Drug Administration  ^a^ Protocol Year is defined as the most recent protocol uploaded on the HMA-EMA catalogue  ^b^ Only outcomes that were captured in at least 4 studies are listed | | | | |

| **Supplementary** **Table 8. Percentage of FDA Studies with Protocol Year Dated^a^ Before and After FDA Guideline Implementation by Most Common Outcomes** | | | | |
| --- | --- | --- | --- | --- |
| **Common Outcomes^b^**  Total Number of Studies | **Pre-2019**  **N=4** | **95% CI** | **Post-2019**  **N=10** | **95% CI** |
|  | **n (%)** |  | **n (%)** |  |
| Stillbirth  N=25 | 3 (75.00%) | [32.56%, 100.00%] | 10 (100.00%) | [100.00%, 100.00%] |
| Congenital malformations (major or minor)  N=24 | 4 (100.00%) | [100.00%, 100.00%] | 7 (70.00%) | [41.60%, 98.40%] |
| Spontaneous abortion  N=22 | 4 (100.00%) | [100.00%, 100.00%] | 7 (70.00%) | [41.60%, 98.40%] |
| Preterm birth or labor  N=21 | 3 (75.00%) | [32.56%, 100.00%] | 9 (90.00%) | [71.41%, 100.00%] |
| Small for gestational age or fetal growth restriction  N=21 | 3 (75.00%) | [32.56%, 100.00%] | 10 (100.00%) | [100.00%, 100.00%] |
| Elective termination  N=15 | 4 (100.00%) | [100.00%, 100.00%] | 4 (40.00%) | [9.64%, 70.36%] |
| Preeclampsia  N=13 | 0 (0.00%) | [0.00%, 0.00%] | 9 (90.00%) | [71.41%, 100.00%] |
| Infant growth and development  N=11 | 3 (75.00%) | [32.56%, 100.00%] | 4 (40.00%) | [9.64%, 70.36%] |
| Gestational hypertension  N=8 | 0 (0.00%) | [0.00%, 0.00%] | 6 (60.00%) | [29.64%, 90.36%] |
| Gestational diabetes  N=8 | 0 (0.00%) | [0.00%, 0.00%] | 5 (50.00%) | [19.01%, 80.99%] |
| Eclampsia  N=7 | 0 (0.00%) | [0.00%, 0.00%] | 7 (70.00%) | [41.60%, 98.40%] |
| Caesarean delivery  N=7 | 1 (25.00%) | [0.00%, 67.44%] | 2 (20.00%) | [0.00%, 44.79%] |
| Low birth weight  N=7 | 1 (25.00%) | [0.00%, 67.44%] | 3 (30.00%) | [1.60%, 58.40%] |
| Infant/neonatal Death  N=7 | 0 (0.00%) | [0.00%, 0.00%] | 4 (40.00%) | [9.64%, 70.36%] |
| Apgar score  N=5 | 2 (50.00%) | [1.00%, 99.00%] | 0 (0.00%) | [0.00%, 0.00%] |
| Admission to NICU  N=4 | 2 (50.00%) | [1.00%, 99.00%] | 2 (20.00%) | [0.00%, 44.79%] |
| Infant/neonatal infections or other illness  N=4 | 2 (50.00%) | [1.00%, 99.00%] | 2 (20.00%) | [0.00%, 44.79%] |
| Abbreviations: FDA = Food and Drug Administration  ^a^ Protocol year is defined as the most recent uploaded publicly available protocol on the HMA-EMA catalogue  ^b^ Only outcomes that were captured in at least 4 studies are listed | | | | |

1. References

1. Evolocumab Pregnancy Exposure Registry: An OTIS Pregnancy Surveillance Study (20150338) [Internet]. 01 Mar2019 [cited 2025 Jul 18]. Report No. Available from: https://catalogues.ema.europa.eu/node/2471/administrative-details

2. Non-interventional Post-Authorisation Safety Study of Burosumab in the Treatment of Children >1 year of age, Adolescents and Adults with X-Linked Hypophosphataemia (XLH PASS) [Internet]. 2021 Oct [cited 2025 Aug 6]. Report No. Available from: https://catalogues.ema.europa.eu/system/files/2024-05/XLH%20Registry%20Protocol%20Amendment%203%20with%20embedded%20PASS_27Oct2021_clean.pdf

3. Retrospective Cohort Study of Pregnancy Outcomes in Women Exposed to Rimegepant During Pregnancy [Internet]. 2022 Aug [cited 2025 Jul 18]. Report No. Available from: https://catalogues.ema.europa.eu/node/2991/administrative-details

4. Safety of Paxlovid During Pregnancy [Internet]. 2023 Jun [cited 2025 Jul 18]. Report No. Available from: https://catalogues.ema.europa.eu/node/3521/administrative-details

5. A Non-Interventional Multi-Database Post-Authorisation Study to Assess Pregnancy-Related Safety Data from Women with Severe Asthma Exposed to Tezepelumab (TREATY) [Internet]. 2023 Jul [cited 2025 Jul 18]. Report No. Available from: https://catalogues.ema.europa.eu/node/4077/administrative-details

6. TG1101-RMS404 [Internet]. 2024 Mar [cited 2025 Jul 18]. Report No. Available from: https://catalogues.ema.europa.eu/node/4513/administrative-details

7. Prospective, Registry-Based Observational Cohort Study of Zavegepant Safety in Pregnancy [Internet]. 2024 Sep [cited 2025 Jul 18]. Report No. Available from: https://catalogues.ema.europa.eu/node/4236/administrative-details

8. A Post-marketing, Observational, Descriptive Study to Assess the Risk Associated With Pregnancy, the Maternal Complications and Adverse Effects on the Developing Fetus, Neonate, and Infant Among Individuals Exposed to Omaveloxolone During Pregnancy and/or Lactation [Internet]. 2024 Jun [cited 2025 Jul 18]. Report No. Available from: https://catalogues.ema.europa.eu/node/4228/administrative-details

9. A Post-Authorisation Safety Study (PASS) of ABRYSVO (Respiratory Syncytial Virus Stabilised Prefusion Subunit Vaccine) in Pregnant Women and their Offspring in a Real World Setting in Europe and UK (C3671026) [Internet]. 2024 Aug [cited 2025 Jul 18]. Report No. Available from: https://catalogues.ema.europa.eu/node/4300/administrative-details

10. A Post-Marketing Safety Study using a Pregnancy Registry to Evaluate the Safety of Respiratory Syncytial Virus Vaccine (ABRYSVO^TM^) Exposure During Pregnancy (C3671041) [Internet]. 2024 May [cited 2025 Jul 18]. Report No. Available from: https://catalogues.ema.europa.eu/node/4080/administrative-details

11. VAC4EU Postauthorisation Safety Study of BIMERVAX® Vaccine in Europe [Internet]. 2024 Jan [cited 2025 Jul 18]. Report No. Available from: https://catalogues.ema.europa.eu/node/4222/administrative-details

12. Observational study of exposure to baricitinib during pregnancy in US-based administrative claims data (I4V-MC-B036) [Internet]. 2025 Jan [cited 2025 Jul 18]. Report No. Available from: https://catalogues.ema.europa.eu/node/3469/administrative-details

13. Observational Cohort Study of Zavegepant Safety in Pregnancy within a US Claims Database [Internet]. 2025 May [cited 2025 Jul 18]. Report No. Available from: https://catalogues.ema.europa.eu/node/4309/administrative-details

14. Prospective, Registry-Based Observational Cohort Study of Ritlecitinib Safety in Pregnancy [Internet]. 2025 Feb. Report No. Available from: https://catalogues.ema.europa.eu/node/4197/administrative-details

15. A Rapid Surveillance and Cohort Post-Marketing Safety Study to Evaluate the Safety of Respiratory Syncytial Virus Vaccine (ABRYSVO^TM^) Exposure During Pregnancy in the United States (C3671027) [Internet]. 2025 Mar [cited 2025 Jul 18]. Report No. Available from: https://catalogues.ema.europa.eu/node/4016/administrative-details

16. An Observational Study to Evaluate the Long-term Safety of Ivacaftor in Patients With Cystic Fibrosis [Internet]. 2013 May [cited 2025 Jul 21]. Report No. Available from: https://catalogues.ema.europa.eu/node/2293/administrative-details

17. Pregnancy Registry to collect Long-Term Safety Data from Women treated with HyQvia [Internet]. 2015 Oct [cited 2025 Jul 18]. Report No. Available from: https://catalogues.ema.europa.eu/node/3040/administrative-details

18. Encruse Ellipta Drug Use Investigation (201450) [Internet]. 2015 Apr [cited 2025 Jul 18]. Report No. Available from: https://catalogues.ema.europa.eu/node/2704/administrative-details

19. International LEMTRADA Pregnancy Exposure Cohort in Multiple Sclerosis (OBS13436) [Internet]. 2016 Nov [cited 2025 Jul 18]. Report No. Available from: https://catalogues.ema.europa.eu/node/3510/administrative-details

20. NN304-4016 An international non-interventional prospective cohort study to evaluate the safety of treatment with Levemir® (insulin detemir) in pregnant women with diabetes mellitus [Internet]. 2017 Sep [cited 2025 Jul 21]. Report No. Available from: https://catalogues.ema.europa.eu/node/2387/administrative-details

21. MLN-0002_401: Entyvio (vedolizumab) long-term safety study: An international observational prospective cohort study comparing vedolizumab to other biologic agents in patients with ulcerative colitis or Crohn’s Disease (Entyvio PASS study) [Internet]. 2017 Oct [cited 2025 Jul 21]. Report No. Available from: https://catalogues.ema.europa.eu/node/3234/administrative-details

22. European Post-Authorization Registry for RAVICTI® (glycerol phenylbutyrate) Oral Liquid in Partnership with the European Registry and Network for Intoxication Type Metabolic Diseases (E-IMD) (HZNP-RAV-401) [Internet]. 2018 Apr [cited 2025 Aug 6]. Report No. Available from: https://catalogues.ema.europa.eu/sites/default/files/document_files/V5%200_RAVICTI-EU-Registry_protocol__26%20Apr%202018_Final%20Signed_Complete_Redacted%20%282%29.pdf

23. A Population-based Cohort Study of Pregabalin to Characterize Pregnancy Outcomes [Internet]. 2018 Nov [cited 2025 Jul 21]. Report No. Available from: https://catalogues.ema.europa.eu/node/2360/administrative-details

24. An International Pregnancy Exposure registry of Women With Multiple Sclerosis (MS) exposed to Teriflunomide (OBS12751) [Internet]. 2018 Jun [cited 2025 Jul 21]. Report No. Available from: https://catalogues.ema.europa.eu/node/2761/administrative-details

25. Utilisation Patterns and Real-World Effects of Tezacaftor and Ivacaftor Combination Therapy (TEZ/IVA) in Patients With Cystic Fibrosis (CF) [Internet]. 2019 Mar [cited 2025 Jul 21]. Report No. Available from: https://catalogues.ema.europa.eu/node/2475/administrative-details

26. Post-Marketing surveillance for the safety of GARDASIL® and GARDASIL®9 in a cohort of Chinese women [Internet]. 2019 Nov [cited 2025 Jul 21]. Report No. Available from: https://catalogues.ema.europa.eu/node/3119/administrative-details

27. A Population-based Study of the Safety of Gabapentin Use During Pregnancy [Internet]. 2020 Oct [cited 2025 Jul 21]. Report No. Available from: https://catalogues.ema.europa.eu/node/3502/administrative-details

28. A descriptive study examining abortion-related complications following reported off-label use of misoprostol (alone or in combination with diclofenac) to induce abortion in Italy. (A6841002) [Internet]. 2020 Jun [cited 2025 Jul 18]. Report No. Available from: https://catalogues.ema.europa.eu/node/2728/administrative-details

29. A Phase IV Non-Interventional Enhanced Active Surveillance Study of Adults Vaccinated with AZD1222 [Internet]. 2021 May [cited 2025 Jul 18]. Report No. Available from: https://catalogues.ema.europa.eu/node/3074/administrative-details

30. A non-interventional post-authorization safety study (NI-PASS) of outcomes associated with the use of tacrolimus around conception, or during pregnancy or lactation using data from Transplant Pregnancy Registry International (TPRI) [Internet]. 2021 Jul [cited 2025 Jul 18]. Report No. Available from: https://catalogues.ema.europa.eu/node/3136/administrative-details
